# Cartography of opportunistic pathogens and antibiotic resistance genes in a tertiary hospital environment

**DOI:** 10.1038/s41591-020-0894-4

**Published:** 2020-06-08

**Authors:** Kern Rei Chng, Chenhao Li, Denis Bertrand, Amanda Hui Qi Ng, Junmei Samantha Kwah, Hwee Meng Low, Chengxuan Tong, Maanasa Natrajan, Michael Hongjie Zhang, Licheng Xu, Karrie Kwan Ki Ko, Eliza Xin Pei Ho, Tamar V. Av-Shalom, Jeanette Woon Pei Teo, Chiea Chuen Khor, MetaSUB Consortium, David Danko, Daniela Bezdan, Ebrahim Afshinnekoo, Sofia Ahsanuddin, Chandrima Bhattacharya, Daniel J. Butler, Kern Rei Chng, Francesca De Filippis, Jochen Hecht, Andre Kahles, Mikhail Karasikov, Nikos C. Kyrpides, Marcus H. Y. Leung, Dmitry Meleshko, Harun Mustafa, Beth Mutai, Russell Y. Neches, Amanda Ng, Marina Nieto-Caballero, Olga Nikolayeva, Tatyana Nikolayeva, Eileen Png, Jorge L. Sanchez, Heba Shaaban, Maria A. Sierra, Xinzhao Tong, Ben Young, Josue Alicea, Malay Bhattacharyya, Ran Blekhman, Eduardo Castro-Nallar, Ana M. Cañas, Aspassia D. Chatziefthimiou, Robert W. Crawford, Youping Deng, Christelle Desnues, Emmanuel Dias-Neto, Daisy Donnellan, Marius Dybwad, Eran Elhaik, Danilo Ercolini, Alina Frolova, Alexandra B. Graf, David C. Green, Iman Hajirasouliha, Mark Hernandez, Gregorio Iraola, Soojin Jang, Angela Jones, Frank J. Kelly, Kaymisha Knights, Paweł P. Łabaj, Patrick K. H. Lee, Levy Shawn, Per Ljungdahl, Abigail Lyons, Gabriella Mason-Buck, Ken McGrath, Emmanuel F. Mongodin, Milton Ozorio Moraes, Niranjan Nagarajan, Houtan Noushmehr, Manuela Oliveira, Stephan Ossowski, Olayinka O. Osuolale, Orhan Özcan, David Paez-Espino, Nicolas Rascovan, Hugues Richard, Gunnar Rätsch, Lynn M. Schriml, Torsten Semmler, Osman U. Sezerman, Leming Shi, Le Huu Song, Haruo Suzuki, Denise Syndercombe Court, Dominique Thomas, Scott W. Tighe, Klas I. Udekwu, Juan A. Ugalde, Brandon Valentine, Dimitar I. Vassilev, Elena Vayndorf, Thirumalaisamy P. Velavan, María M. Zambrano, Jifeng Zhu, Sibo Zhu, Christopher E. Mason, Swaine L. Chen, Christopher E. Mason, Oon Tek Ng, Kalisvar Marimuthu, Brenda Ang, Niranjan Nagarajan

**Affiliations:** 1 Computational and Systems Biology, Genome Institute of Singapore, Singapore, Singapore; 2 Information Systems Technology and Design, Singapore University of Technology and Design, Singapore, Singapore; 3 Department of Microbiology, Singapore General Hospital, Singapore, Singapore; 4 Department of Molecular Pathology, Singapore General Hospital, Singapore, Singapore; 5 Duke-NUS Graduate Medical School, Singapore, Singapore; 6 Department of Laboratory Medicine, National University Hospital, Singapore, Singapore; 7 Department of Physiology and Biophysics, Weill Cornell Medicine, New York, NY USA; 8 National Centre for Infectious Diseases, Singapore, Singapore; 9 Department of Infectious Diseases, Tan Tock Seng Hospital, Singapore, Singapore; 10 Lee Kong Chian School of Medicine, Nanyang Technological University, Singapore, Singapore; 11 Yong Loo Lin School of Medicine, National University of Singapore, Singapore, Singapore; 12 Weill Cornell Medicine, New York, NY USA; 13 The Bin Talal Bin Abdulaziz Alsaud Institute for Computational Biomedicine, New York, NY USA; 14 Icahn School of Medicine at Mount Sinai, New York, NY USA; 15 Genome Institute of Singapore, Singapore, Singapore; 16 University of Naples Federico II, Naples, Italy; 17 The Barcelona Institute of Science and Technology, Barcelona, Spain; 18 ETH Zurich, Zurich, Switzerland; 19 Joint Genome Institute, Walnut Creek, CA USA; 20 City University of Hong Kong, Hong Kong SAR, China; 21 Kenya Medical Research Institute/Medical Research Directorate-Africa, Kisumu, Kenya; 22 University of Colorado Boulder, Boulder, CO USA; 23 Indian Statistical Institute, Kolkata, India; 24 University of Minnesota, Minneapolis, MN USA; 25 Universidad Andrés Bello, Santiago, Chile; 26 Weill Cornell Medicine—Qatar, Doha, Qatar; 27 California State University Sacramento, Sacramento, CA USA; 28 University of Hawaii, Honolulu, HI USA; 29 Aix-Marseille Université, Marseille, France; 30 A.C.Camargo Cancer Center, São Paulo, Brazil; 31 Norwegian Defence Research Establishment, Kjeller, Norway; 32 University of Sheffield, Sheffield, UK; 33 Institute of Molecular Biology and Genetics of National Academy of Sciences of Ukraine, Kyiv, Ukraine; 34 University of Applied Sciences FH-Campus Wien, Vienna, Austria; 35 King’s College London, London, UK; 36 Institut Pasteur de Montevideo, Montevideo, Uruguay; 37 Institut Pasteur Korea, Seongnam, South Korea; 38 Corporación Corpogen, Bogotá, Colombia; 39 Jagiellonian University, Kraków, Poland; 40 HudsonAlpha Institute for Biotechnology, Huntsville, AL USA; 41 Stockholm University, Stockholm, Sweden; 42 Microba, Brisbane, Australia; 43 University of Maryland School of Medicine, Baltimore, MD USA; 44 Fundação Oswaldo Cruz Laboratório de Hanseníase, Rio de Janeiro, Brazil; 45 Ribeirão Preto Medical School University of São Paulo, São Paulo, Brazil; 46 Instituto de Patologia e Imunologia Molecular da Universidade do Porto, Porto, Portugal; 47 University of Tübingen, Tübingen, Germany; 48 Elizade University, Ondo State, Nigeria; 49 Acibadem Mehmet Ali Aydinlar University, Istanbul, Turkey; 50 Sorbonne University, Paris, France; 51 Robert Koch Institute, Berlin, Germany; 52 Fudan University, Shanghai, China; 53 Vietnamese-German Center of Excellence, Hanoi, Vietnam; 54 Keio University, Fujisawa, Japan; 55 University of Vermont, Burlington, VT USA; 56 Millennium Initiative for Collaborative Research on Bacterial Resistance, Santiago, Chile; 57 Sofia University, Sofia, Bulgaria; 58 University of Alaska Fairbanks, Fairbanks, AK USA; 59 Univeristätsklinikum Tübingen, Tübingen, Germany

**Keywords:** Microbial genetics, Disease prevention

## Abstract

Although disinfection is key to infection control, the colonization patterns and resistomes of hospital-environment microbes remain underexplored. We report the first extensive genomic characterization of microbiomes, pathogens and antibiotic resistance cassettes in a tertiary-care hospital, from repeated sampling (up to 1.5 years apart) of 179 sites associated with 45 beds. Deep shotgun metagenomics unveiled distinct ecological niches of microbes and antibiotic resistance genes characterized by biofilm-forming and human-microbiome-influenced environments with corresponding patterns of spatiotemporal divergence. Quasi-metagenomics with nanopore sequencing provided thousands of high-contiguity genomes, phage and plasmid sequences (>60% novel), enabling characterization of resistome and mobilome diversity and dynamic architectures in hospital environments. Phylogenetics identified multidrug-resistant strains as being widely distributed and stably colonizing across sites. Comparisons with clinical isolates indicated that such microbes can persist in hospitals for extended periods (>8 years), to opportunistically infect patients. These findings highlight the importance of characterizing antibiotic resistance reservoirs in hospitals and establish the feasibility of systematic surveys to target resources for preventing infections.

## Abstract (web summary)

Spatiotemporal characterization of microbial diversity and antibiotic resistance in a tertiary-care hospital reveals broad distribution and persistence of antibiotic-resistant organisms that could cause opportunistic infections in a healthcare setting.

## Main

The global epidemic of antibiotic resistance has refocused attention on infection prevention and control in hospitals^1^. It is estimated that if the spread of antibiotic resistance grows unchecked, it will cause millions of deaths worldwide, with an economic impact of more than US$100 trillion by 2050 (ref. ^2^). Hospital-acquired infections (HAIs) pose a high healthcare burden in both developed and developing countries^3^. US estimates highlight that 1 in 25 acute-care patients have active HAIs daily (721,800 HAIs each year), with 11.5% of patients dying during hospitalization^4^. The problem of HAIs is further compounded by the global spread of multidrug-resistant organisms (MDROs), complicating infection management, limiting therapy options and resulting in poorer outcomes^5^. The risk of HAIs can be mitigated through good infection prevention practice, with hand hygiene advocated as an important strategy to limit spread between patients and medical staff^6^.

In addition to human-to-human transfer, the hospital environment is another key transmission network node, with mounting evidence that it harbors opportunistic antibiotic-resistant pathogens contributing to HAIs^7^. Reinforced environmental cleaning measures have shown effectiveness in reducing HAIs^8^. The microbial ecology and uncharacterized genetic reservoirs of hospital environments are thus of interest for both infection epidemiology and microbiology. For example, transmission and recombination profiles of antibiotic resistance genes (ARGs) in hospitals remain largely unknown and could help gauge risk for emergence of novel resistance combinations. Similarly, comparative genomics of hospital-adapted and epidemic strains could identify the source of outbreaks and inform infection control. While large-scale surveillance holds promise to reveal clinical and biological insights pertaining to the hospital microbiome as a reservoir of pathogens and ARGs, significant technological challenges remain. Traditionally, efforts to survey the hospital environment have focused on culture-based isolation of specific pathogens, with each isolate individually characterized via functional profiling, genotyping and/or whole-genome sequencing^9–11^. This is laborious, is prone to isolation bias and precludes insights into overall community structure and how that interacts with the built environment to impact HAIs^12^.

The development of metagenomics enables profiling of overall community structure, characterizing individual microbes without isolation, and represents a scalable, high-throughput method for surveying hospital environments^13^. This has been leveraged through 16S rRNA sequencing in early studies of bacterial diversity, particularly in intensive care units (ICUs)^14^. Lax et al. used this approach to extensively characterize microbial ecology, colonization and succession in a newly built hospital^15^. Using bioinformatics approaches, the authors identified ecological signatures of bacterial exchange between the environment, patients and healthcare workers. However, 16S rRNA sequencing precludes detailed analysis of nosocomial strains, resistomes, metabolic pathways and transmission of pathogenomes^16^. Brooks et al. used Illumina shotgun metagenomics to characterize strain polymorphisms and relatedness of pathogens in low-diversity neonatal ICU environments^17^. Several limitations remain for the use of shotgun metagenomics in general, including low biomass, the presence of multiple strains and pathogens at low abundances, inaccuracies in strain-level analysis^18^, and shortcomings of short reads for assembling high-contiguity, strain-resolved genomes for detailed genetic analyses^19^.

The availability of long-read sequencing presents new opportunities and challenges for pathogenome and resistome monitoring^20^. Here, we combined extensive short-read shotgun metagenomics of multiple sites, wards and time points (*n* = 428) with enrichment and nanopore sequencing of antibiotic-resistant mixed cultures (*n* = 1,661) to provide the most extensive genetic characterization of hospital environments to date. The combination of metagenomic surveys (short-read based) with detailed genomic analysis of nosocomial strains (long-read based) is ideal for studying distribution, abundance and turnover patterns of pathogens and ARGs. Nanopore metagenomics enabled the generation of thousands of high-contiguity genomes (*n* = 2,347), phage sequences (*n* = 1,693) and closed plasmid sequences (*n* = 5,910), revealing substantial uncharacterized genetic diversity (>60% novel). These were harbored in distinct ecological niches characterized by biofilm-forming and human-microbiome-associated bacteria, with divergent patterns of spatiotemporal variation. Phylogenetic analysis highlighted that MDROs are more likely to be widely distributed and stably colonizing across hospital sites. Analysis of ARG combinations and phage and plasmid architectures revealed the dynamic nature of hospital-environment resistomes. Genomic comparisons with patient isolates across multiple species indicated that MDROs persist in the hospital environment for extended periods (>8 years) to opportunistically infect patients. These findings underscore the importance of characterizing hospital microbiomes to understand niches and genetic reservoirs, the need for improved disinfection methods and the feasibility of large-scale genomic surveys to inform infection control.

## Results

### Hospital-environment microbiomes offer distinct ecological niches for opportunistic pathogens and ARGs

**Fig. 1:**
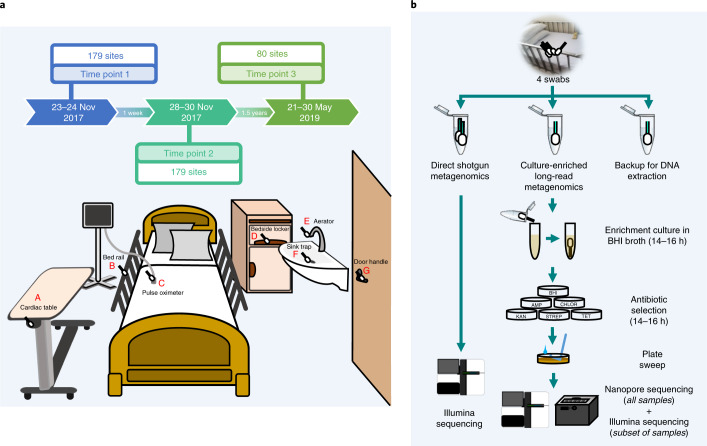
Overview of sampling sites and analysis workflow. **a**, Diagram showing the various sites that were sampled, including cardiac tables (A), bed rails (B), pulse oximeters (C), bedside lockers (D), sink aerators (E), sink traps (F) and door handles (G). Each ward (MDRO and standard) had five beds (sites A–D individually sampled), one sink (E,F) and no doors, while isolation rooms had one bed (A–D), one sink (E,F) and a door (G). **b**, Diagram showing the analysis workflow for the four swabs that were collected from each site in terms of culturing, DNA extraction and sequencing. Samples from each site were analyzed with shotgun metagenomic sequencing on the Illumina platform and multiple (*n* = 6) culture-enriched quasi-metagenomics on a GridION system.

**Fig. 2:**
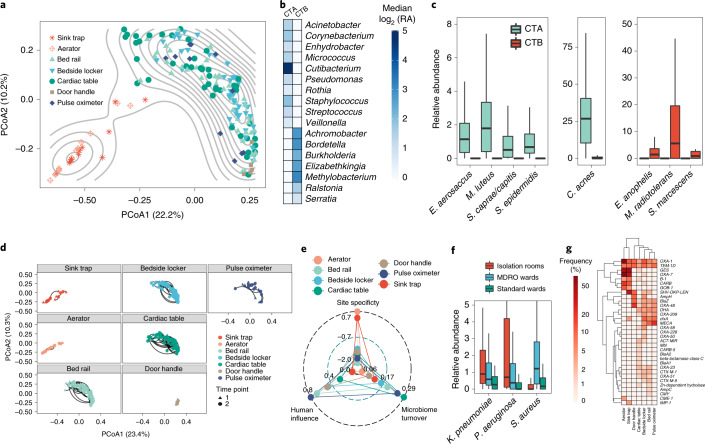
Distinct ecological niches in the hospital environment for microbes and ARGs. **a**, PCoA plot based on genus-level Bray–Curtis dissimilarity of taxonomic profiles (*n* = 176 independent samples, time point 1) indicating two distinct community types (denoted as CTA and CTB) for microbiomes from the hospital environment. **b**, Heat map showing relative abundances (log-scale, log_2_ (RA)) for differentially abundant genera between CTA and CTB (false discovery rate (FDR)-adjusted two-sided Wilcoxon *P* value < 0.01; *n* = 151 and 25 independent samples for CTA and CTB sites, respectively). **c**, Box plots showing relative abundances for differentially abundant species between CTA and CTB (FDR-adjusted two-sided Wilcoxon *P* value < 0.01; *n* = 151 and 25 independent samples for CTA and CTB sites, respectively). In the box plots, the center line is the median; box limits are the upper and lower quartiles; and whiskers are 1.5 times the interquartile range (outlier points are not included in the visualization). **d**, PCoA plots (genus-level Bray–Curtis dissimilarity) showing variation in environmental microbiomes over time (lines connect two time points, 1 week apart) for different sites (*n* = 24, 26, 90, 90, 90, 10 and 22 independent samples for sink traps, aerators, bed rails, bedside lockers, cardiac tables, door handles and pulse oximeters, respectively). **e**, Radar plot showing the microbiome turnover index (fraction of taxa that are gained or lost across time points), human influence index (fraction of human reads) and site specificity index (uniqueness of site-specific taxonomic composition in relation to physically proximal sites). A positive site-specificity index indicates a stronger site-specific microbiome composition signature. **f**, Box plots showing relative abundances of common nosocomial pathogens that were differentially abundant across ward types in sites with high human contact (FDR-adjusted Kruskal–Wallis test, *P* value < 0.01; *n* = 48, 128 and 128 independent samples for isolation rooms, MDRO wards and standard wards, respectively). In the box plots, the center line is the median; box limits are the upper and lower quartiles; and whiskers are 1.5 times the interquartile range (outlier points are not included in the visualization). **g**, Heat map depicting the frequency of detection for beta-lactamases at different sites in hospital wards. Multiple carbapenemases and the *mecA* gene were detected as part of the resistomes that were primarily defined by the community types (CTA and CTB).

A diverse set of sites (*n* = 7) of concern for infection control^21,22^ and different room types distributed around the building (5 single-bed isolation rooms together with 4 MDRO and 4 standard five-bed wards) were selected for initial sampling at two time points (1 week apart) of a tertiary-care hospital in Singapore (45 beds (4% of total), 179 sites, 358 samples; Fig. 1a and Supplementary Data 1). Illumina shotgun metagenomics (2 × 101 bp) was used to deeply characterize each sample (average = 30 × 10^6^ reads; 3 of 358 libraries were excluded due to low biomass) to obtain taxonomic profiles and resistomes (Fig. 1b, Supplementary Data 2 and Methods). Controls, spike-ins and validation experiments were used to assess and account for the impact of kit contaminants on low-biomass samples^23^, with likely contaminants identified using batch and correlation analysis^23^ and filtered from profiles (Supplementary Note 1, Supplementary Data 2 and Methods). Taxonomic profiles were visualized using a principal-coordinates analysis (PCoA) plot to identify two distinct microbial community configurations in the hospital environment (Fig. 2a). While community type A (CTA) sites were more taxonomically diverse (Wilcoxon *P* value < 10^−3^; Supplementary Fig. 1) and largely high-touch surfaces with frequent contact from patients and healthcare workers^24^, community type B (CTB) represents sites of increasing concern for infection control for their propensity to harbor MDROs^10,21,25^. Joint analysis of these community types helped to identify key taxonomic features that differentiate them, including several human-microbiome-associated genera (for example, *Cutibacterium*) and aquatic and terrestrial environment-associated genera (for example, *Achromobacter*) in CTA and CTB, respectively, although not all genera could be defined in these terms (for example, *Pseudomonas*, *Acinetobacter* and *Ralstonia*; Fig. 2b). At the species level, key differences included enrichment of common skin bacteria (for example, *Cutibacterium acnes* and *Staphylococcus epidermidis*) and biofilm-associated organisms in hospitals (for example, *Elizabethkingia anophelis* and *Serratia marcescens*) in CTA and CTB sites, respectively, although their occurrences were not mutually exclusive, indicating shared influences (Fig. 2c). The comparison of hospital microbiome CTA and CTB sites to similar sites in an indoor office environment (*n* = 30, office; Supplementary Data 1 and Methods) and other high-touch environmental microbiomes^26^ (*n* = 99, MetaSUB Singapore; Supplementary Data 1) further highlighted the distinctness of hospital environments and community types (Supplementary Fig. 2) and the corresponding utility as an organizing principle for studying clinical impact^14,27^.

Microbiomes associated with the community types exhibited varying stability across the sampled time points, with CTA sites demonstrating larger fluctuations (except door handles; Wilcoxon *P* value < 10^−3^; Fig. 2d). Microbial profiles diverged with distance (within a bed, within wards and across wards) and time (1 week apart), with temporal variability within a week being lower than spatial variability within a ward (Wilcoxon *P* value < 10^−3^; Supplementary Fig. 3a). Analysis of a subset of sites (*n* = 80) resampled at a third time point >1 year later confirmed long-term stability of community types across sites (Supplementary Fig. 3b). Microbial composition of sites is expected to be influenced by several factors, including abiotic conditions (humidity, temperature and surface type), seeding from microbial reservoirs (human or environmental) and exchange across sites. Based on sequencing data, we computed scores to quantify these factors, including a microbiome turnover index (fraction of taxa gained or lost across time points), a human influence index (fraction of human reads) and a site specificity index (uniqueness of site-specific taxonomic composition relative to proximal sites), each of which exhibited significantly correlated trends across time points (Supplementary Fig. 4a). The computed indices reinforce the notion that CTB sites (primarily sink traps and aerators) have stable compositions (low turnover) based on site-specific biofilm configurations with limited human microbiome seeding (Fig. 2e). CTA sites showed higher human influence (Wilcoxon *P* value < 10^−15^) and microbiome turnover (Wilcoxon *P* value < 10^−4^) indices, although they were not directly correlated, and showed weaker site specificity (Wilcoxon *P* value < 10^−12^), concordant with a model where human activities (patient discharge and admittance events) have a systemic role in shaping site compositions (Fig. 2e). Species that were enriched in CTA sites were also observed in CTB sites (and vice versa) but had higher turnover in these cases (Supplementary Fig. 4b), with some exceptions such as *Siphoviridae*, which had high turnover in both CTA and CTB sites.

Overall, patterns of microbiome variability were consistent across ward types, although isolation rooms exhibited lower variability across time points (Supplementary Fig. 5). In line with Singapore’s MDRO management guidelines^28^, patients colonized with carbapenem-resistant Enterobacteriaceae (CRE; for example, *Klebsiella* *pneumoniae*) were typically in single-bed isolation rooms, while patients with methicillin-resistant *Staphylococcus aureus* (MRSA) were in MDRO wards. An analysis of differentially abundant nosocomial pathogens (curated from https://www.cdc.gov/hai/organisms/organisms.html and publications^4,29^) detected across ward types identified *K. pneumoniae* and *S. aureus* as being enriched in CTA sites for isolation rooms and MDRO wards, respectively, providing further evidence for the influence of patient microbiomes on CTA sites (Fig. 2f). Consistent with observed taxonomic differences, CTA and CTB sites harbored distinct complements of ARGs in their resistomes (Fig. 2g and Supplementary Fig. 6). While some ARGs were frequently detected in CTB sites (for example, *ges* and *oxa-7*; Fig. 2g), CTA sites carried a wider diversity of ARGs at lower frequencies. Despite recent focus on CTB sites as ARG reservoirs^10,25^, some clinically important ARGs such as *oxa-23* (encoding a carbapenemase) and *mecA* (methicillin resistance) were more frequently found in CTA sites, while genes such as *imp-1* (carbapenemase) and *cme-1* (extended-spectrum beta-lactamase) were more common in CTB sites. Different sites also exhibited distinct resistome patterns—for example, specific tetracycline (*tetC*) and macrolide (*mphE*) resistance genes were highly enriched only in aerators, while vancomycin resistance genes were only observed in bedside lockers and on bed rails—highlighting the importance of considering site- and ward-specific patterns for infection control and drug resistance mitigation strategies (Supplementary Fig. 6). While a higher proportion of ARGs was consistently detected across time points in CTB sites compared to CTA sites (Supplementary Fig. 7a), overall, hospital microbiomes exhibited significantly higher abundance (>3-fold versus MetaSUB Singapore and >12-fold versus office sites, Wilcoxon *P* value < 10^−15^ for both comparisons; Supplementary Fig. 7b) and higher diversity (Wilcoxon *P* value < 10^−15^; Supplementary Fig. 7c) of ARGs compared to other high-touch urban environmental microbiomes. Even though the presence of ARGs does not always translate to resistance phenotypes, these results further underscore the distinctness of hospital microbiomes as ARG reservoirs^30^.

### Quasi-metagenomics with nanopore sequencing reveals distribution of multidrug-resistant opportunistic pathogens in the hospital environment

**Fig. 3:**
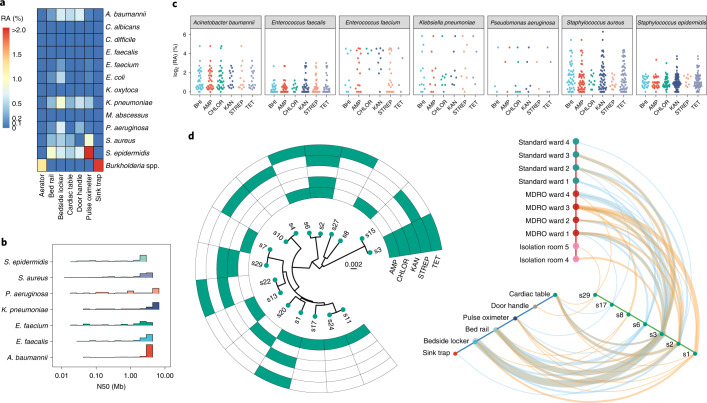
Genome-resolved characterization of nosocomial multidrug-resistant strains that spread and persist at low relative abundances in the hospital environment. **a**, Heat map displaying the distinct median relative abundance (RA) of common nosocomial pathogens at different sites (*n* = 26, 90, 90, 90, 10, 22 and 24 for aerators, bed rails, bedside lockers, cardiac tables, door handles, pulse oximeters and sink traps, respectively) in hospital environments (PERMANOVA *P* value < 0.001). **b**, Distribution of assembly contiguity statistics (N50 = fragment size such that more than 50% of the genome is in longer sequences) for common nosocomial pathogens, highlighting the high genomic contiguity that was obtained (median N50 > 1 Mb). **c**, Dot plots highlighting that genomes can be rapidly obtained for several nosocomial pathogens despite their low relative abundances in corresponding environmental microbiomes (*y* axis) through an enrichment and long-read metagenomic sequencing-based protocol. Represented species are associated with more than 20 genome drafts in the overall database of 2,347 genomes. **d**, Left, phylogenetic relationships of *S. aureus* derivative clusters (>99.99% ANI; each node represents the consensus genome for the cluster) detected in the hospital environment together with their antibiotic resistance profiles. The scale bar represents the number of substitutions per site in the core alignment. Right, hive map representation showing localization of *S. aureus* clusters that spread (detected at two or more locations) and/or persist (detected in time points 1 and 2) in the hospital environment. The colored lines represent occurrence at time point 1 (orange) and time point 2 (blue); line thickness represents the number of instances of such occurrences. Note that multidrug-resistant strains such as s3, s2 and s1 tend to be more widely distributed and persistent in the hospital environment.

Based on Illumina metagenomic profiles, we noted that nosocomial pathogens were generally present at low relative abundances (median relative abundance < 0.5%; Fig. 3a) in hospital environments (even though this was higher than in other urban sites; Supplementary Fig. 7d), precluding detailed genomic characterization of transmission patterns, ARG combinations and plasmids. The distribution of common pathogens exhibited site-specific patterns (PERMANOVA *P* value < 0.001; Fig. 3a), in agreement with the distinct niches observed in hospital environments (Fig. 2a–c), and indicated that enrichment cultures could capture a diverse set of species. We exploited this observation to use a culturing, antibiotic selection (five antibiotics) and metagenomic nanopore sequencing approach (Fig. 1b) to obtain a large database of high-contiguity assemblies (*n* = 2,347) from the hospital environment (median N50 > 1 Mb; Fig. 3b, Supplementary Data 3 and Methods), expanding substantially on genomic resources established by previous studies^10,11^. Overall, a large percentage of sites led to viable cultures (>95%), with antibiotic selection resulting in growth in >80% of plates (1,495 of 1,790) and >42% of sites resulting in cultures for all five antibiotics. Control swabs led to no cultures (0 of 10), confirming that cultures were not likely due to contamination (Methods), and further testing of isolates confirmed that the vast majority of strains in the cultures were likely to be antibiotic resistant (99%; Supplementary Note 2).

DNA was extracted from 1,661 plates and sequenced on a GridION to provide 535 Mb of data on average per sample (median read length > 2.5 kb). Long-read metagenomic assembly enabled the reconstruction of megabase-pair sized contigs (versus average N50 < 5 kb for Illumina assemblies) as the communities were largely simple (Supplementary Fig. 8, Fig. 3b and Methods). Evaluation of these draft genomes based on conserved single-copy genes confirmed that they were of high quality (completeness > 99%, contamination < 0.5%; Methods). In total, we obtained genomes for 69 species from the hospital environment, 40% of which belonged to common pathogens (Methods). Our results confirm the viability of these species in different hospital environments and the ability to enrich them for sequencing and genome reconstruction despite their low abundances in hospital microbiomes (median relative abundance = 0.68%, averaged across species; Fig. 3c). Large-scale homology analysis with public databases^31–33^ also helped to identify 13 (out of >80) species-level clusters (11 different genera including *Bacillus*, *Pseudomonas* and *Staphylococcus*) with no representatives from known species, highlighting recovery of high-quality genomes for novel species using this approach (Methods). Rarefaction analysis showed that >90% of the species and resistance gene diversity (>50% richness) that could be sampled from sites in this study was captured by our sample size (Supplementary Fig. 9), while substantial additional diversity remains to be captured for plasmids and HAI-associated strains (Supplementary Note 3). This confirms the viability of future surveys of ARGs in hospitals with much fewer samples (*n* ≈ 50), making regular surveys feasible, affordable and potentially actionable.

As plasmids and phages serve as an important medium for the evolution and spread of ARGs and emergence of multidrug resistance^34,35^, we characterized corresponding sequences in our genomic database (Methods). In total, we recovered 696 Mb of plasmid sequences (*n* = 5,910 closed and 493 Mb of linear fragments) and 63 Mb of phage sequences (*n* = 1,693, of which 277 are circular), most of which are not present in existing databases for plasmids^36^ or phages^37^ (>90%; 1,505 of 1,588 plasmid clusters and 501 of 557 phage clusters; Methods) despite being commonly distributed in the hospital (Supplementary Fig. 10), highlighting its underexplored genetic diversity. Many closed plasmids were >100 kb long (>9%, *n* = 536), rich in repeats and present at low abundance, impeding characterization using Illumina metagenomics. We noted the presence of several large *mecA*-carrying plasmids that contained antiseptic or disinfectant resistance genes (*qacA* or *qacC*), a combination that is not represented in existing databases^36^ but is in agreement with high biocide resistance for MRSA in clinical settings^38^. One of the plasmids had genes from several additional ARG classes that have not been seen in combination (for example, *dfrC*, *lnuA* and *aac6-Aph2*), highlighting the value of closed sequences for characterization of novel ARG combinations.

The availability of a large collection of highly contiguous plasmid (closed) and chromosomal (megabase-pair contigs) assemblies allowed us to perform genomic relatedness (with environmental and patient strains) and structural (common gene cassettes and exchange across cassettes) analysis. We first analyzed evolutionary relationships between genomes from the hospital, with previously used thresholds of average nucleotide identity (ANI) to define strain-level^39^ (>99.9% ANI), derivative^10^ (>99.99% ANI) and direct-transfer^17^ (>99.999% ANI) genome clusters, and understand their spatiotemporal distribution. For many species, a diverse set of clusters was observed across the hospital (*n* = 6, *Pseudomonas aeruginosa* to *n* = 46, *S. epidermidis*; Fig. 3d and Supplementary Fig. 11). Some genome clusters were frequently detected at multiple sites and ward types in the hospital, and these were also significantly enriched for clusters detected in the first and second time points (Fisher’s exact test *P* value < 1.5 × 10^−9^). Even at the most stringent threshold (direct transfer), a substantial fraction of genomes observed in the third time point (1.5 years later) clustered with genomes from earlier time points for various species (*E. anophelis*: 92%, as few as 5 single nucleotide polymorphisms (SNPs); *S. marcescens*: 20%, 16 SNPs; *Staphylococcus haemolyticus*: 21%, 8 SNPs), emphasizing the stability of environmental pathogenomes.

Overlaying antibiotic resistance information with these patterns, we noted an enrichment of multiantibiotic resistance among strains that were widely distributed through space and time (>2 antibiotics; Fisher’s exact test *P* value < 3 × 10^−8^; Fig. 3d and Supplementary Fig. 12). This was also consistently observed across several common pathogens in the hospital (Fisher’s exact test *P* value: 1.6 × 10^−2^, *S. aureus*; 2.3 × 10^−3^, *S. epidermidis*; 3.7 × 10^−3^, *Enterococcus faecalis*; 5.0 × 10^−2^, *Acinetobacter baumannii*). For a subset of species (*S. aureus*, *S. epidermidis* and *A. baumannii*), we used Illumina sequencing to generate hybrid assemblies and reliably detect derivative and direct-transfer relationships (Methods). Genomes that were related across early time points based on these stringent criteria continued to be significantly enriched for multidrug resistance (binomial test *P* value < 10^−5^, all species and both thresholds) and were also enriched in the third time point (derivative clusters, binomial test *P* value: 0.028, *S*. *epidermidis*; 5.0 × 10^−5^, *S. aureus*), highlighting the presence of stable, viable environmental reservoirs for pathogens and the need to understand the mechanisms contributing to enrichment of multidrug-resistant strains^40,41^.

### Diversity and dynamics of ARG cassettes in the hospital environment

With increasing multidrug resistance, the specific combination of ARGs that is harbored is important to know from a clinical perspective. In hospital environments, little is known about the diversity of ARG combinations and genetic exchange across genomic cassettes and plasmids. Comparing our database of 2,347 high-contiguity genomes and 5,910 closed plasmids against existing databases, we found that 34% of the ARG combinations observed were novel (255 of 752; Supplementary Data 4). Certain ARG combinations have obvious clinical importance, for example, the co-occurrence of *mecA* with *fosB* (fosfomycin resistance) in several environmental *S. aureus* strains, an observation that is concerning given the potential utility of fosfomycin for treating MRSA infections^42^. Notably, we detected Enterobacteriaceae*-*associated genes that can confer resistance to gentamicin (*aac3-IIa*), fosfomycin (*fosA*, *fosA2*) and colistin (*mcr1*), all last-resort antibiotics for CRE infections. Additionally, two Enterobacteriaceae*-*associated plasmids, one carrying *fosA* and the other carrying *mcr1*, were obtained from the same bedside locker, highlighting the potential reservoir for emergence of co-resistance to colistin and fosfomycin. Another Enterobacteriaceae*-*associated plasmid carried a rifampicin resistance gene (*arr*), a telling observation given the growing interest in using rifampicin in combination treatments for a variety of Gram-negative infections, for example, *A. baumannii*^43,44^.

**Fig. 4:**
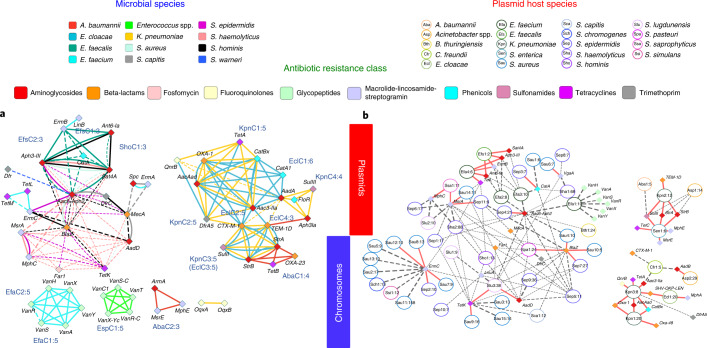
Species distribution and genomic proximity of drug resistance genes in the hospital-environment microbiome. Genomic proximity network and clustering of ARGs based on 2,347 microbial genomes and 5,910 closed plasmid sequences obtained from the hospital environment. **a**, Multigraph of genomic proximity between ARGs. Colored edges indicate gene pairs found <10 kb apart in the genomes for a species (excluding plasmids). Line widths indicate the frequency of occurrence of gene pairs (normalized by count for the rarer gene), and frequencies >80% are marked with solid lines. Solid-line cliques in each species were used to define cassettes and assign names (Supplementary Data 5), and the number after the colon indicates clique size. Genes are colored according to their respective antibiotic classes. **b**, Circles represent different plasmid clusters (95% identity), and their corresponding ARGs are connected by edges and indicated by diamonds. Plasmid nodes were labeled based on a three-letter short form for the host species and assigned a number (for example, Kpn1 for a *K. pneumoniae* plasmid); the number after the colon indicates how many representatives of the plasmid family were observed in the database. Edges are weighted by the frequency at which a gene is present in a plasmid, and frequencies >80% are indicated with red solid lines. Genes and backbones are color coded according to their respective ARG classes and inferred host species for ease of reference.

We next identified common ARG pairs that were in close proximity (<10 kb apart) to determine chromosomal cassettes that may serve as the unit of evolution, co-regulation and ARG exchange (Methods). Chromosomal cassettes were generally small (2–6 genes, average = 3) and specific to a species, although two large cassettes carrying extended-spectrum beta-lactamases were found to overlap for *K. pneumonia* and *Enterobacter cloacae* (KpnC1, KpnC2 and EclC1, EclC2; KpnC3 and EclC3; Fig. 4a and Supplementary Data 5). Selective pressure from rampant use of beta-lactams and plasmid-mediated transmission could have contributed to the sharing of these large cassettes across species. Cassettes for Gram-negative species were larger and more stable (solid lines to genes), while those for Gram-positive species were smaller with many variably present members (dashed lines to genes). The largest shared cassette among Gram-positive species (aminoglycoside-streptothricin resistance; *ant6-Ia*, *sat4A* and *aph3-III*) was in *Enterococcus* and *Staphylococcus* but with no discernible signals of mobile elements^45^. While most genes were stably present in cassettes, with some exceptions (for example, *tetK*, *far1* and *catA*), the exchange of genes across cassettes was rarely observed (for example, *blaZ*), indicating that chromosomal cassettes tend to be relatively fixed.

Performing a similar analysis for closed plasmids, we first clustered them into shared backbones and annotated them for known hosts (identity ≥ 95%; Methods). By analyzing ARGs in this context, we found that many ARGs were variably present in backbones (93 of 143). For ARGs stably found in one backbone, many were variably present in another backbone (19 of 31), highlighting the dynamic nature of ARG combinations from plasmids in the hospital environment (Fig. 4b). Despite this, some ARG combinations were stably present in multiple plasmid backbones, indicating strong selection for coexistence. For example, the genes *strA*, *strB* (streptomycin resistance) and *sulII* (sulfonamide resistance) co-occurred in two distinct backbones (Sen1 and Kpn2, sequence overlap < 54%), likely as a signature from past co-administration of streptomycin and sulfonamides^46,47^. Similarly, while aminoglycoside resistance genes such as *aadD* and *aac6-Aph2* were widely distributed across plasmid backbones, *ant6-Ia* and *aph3-III* were stably shared by two distinct backbones (Efa4 and Efs1, sequence overlap <11%) indicating that they may provide synergistic resistance to aminoglycosides by catalyzing different modifications. Notably, genes that are widely distributed across plasmids (for example, *tetK*, *far1* and *blaZ*) can come together in a novel, clinically relevant backbone (Fig. 4b; Slu3, with 38 sequences in our database), as described for a cytotoxin-producing MRSA strain^48^. While the previously isolated strain was resistant to fusidic acid and tetracycline, but susceptible to erythromycin and clindamycin, we noted the presence of a common plasmid backbone in our database (Sha2 with 88 sequences) that carried a new combination of resistance genes for all four antibiotics (Fig. 4b and Supplementary Data 4). Similarly, we observed that ARGs found in phages, such as *aac6-Aph2* and *far1*, tended to be more widely present (Fig. 4 and Supplementary Fig. 13a), with evidence for recent phage-mediated dissemination of *far1* across *Staphylococcus* species (Supplementary Fig. 13b). In general, ARGs found in plasmids tended to have more ARGs in close proximity (<10 kb apart) in chromosomes than chromosome-exclusive ARGs (Wilcoxon test *P* value = 6 × 10^−7^), characteristic of higher gene mobility and shuffling for plasmid-associated genes. Thus, plasmid backbones seen in the hospital environment likely represent a more plastic framework to generate diverse ARG combinations, many of which are not seen in genomic cassettes (25%) despite strong overlap in the complement of ARGs that they harbor (84% of plasmid genes).

### Hospital-environment strains overlapping with patient isolates are globally disseminated and enriched for multidrug resistance

**Fig. 5:**
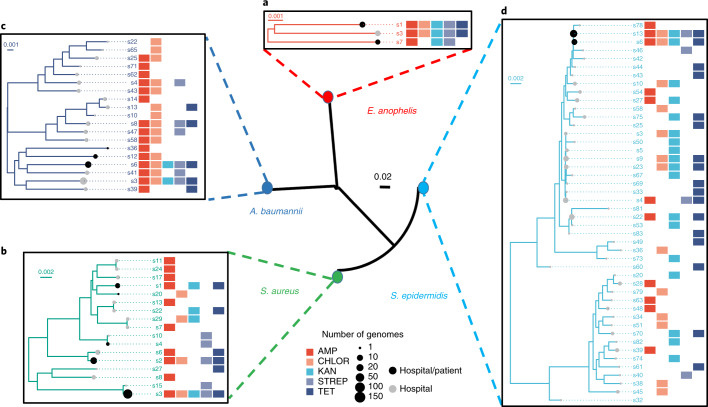
Multispecies analysis of phylogenetic relationships between environmental and patient genomes. Phylogenies depict the evolutionary relationships between derivative clusters (>99.99% ANI), with each node representing the consensus genome for a cluster. **a**, *E. anophelis* from a nosocomial outbreak in Singapore in 2012 and other patient isolates from 2009–2012, **b**, patient-colonizing *S. aureus* from a 2009–2011 surveillance study in Singaporean hospitals, **c**, infectious *A*. *baumannii* isolates from patients in two major Kuwaiti hospitals and Singaporean patient isolates, and **d**, recent globally disseminated multidrug-resistant *S*. *epidermidis* lineages, together with environmental genomes for corresponding species from this study. While **a** and **b** highlight the close relationships between the strains circulating in Singaporean hospitals up to 8 years apart, **c** and **d** reveal the global dissemination of several lineages. The matrices next to the trees indicate the antibiotic resistance profiles for corresponding derivative clusters. Scale bars depict the number of substitutions for each site in the core alignment. For all species tested, clusters shared between environmental and patient genomes were enriched for multidrug resistance.

The availability of a large database of genomes from many species in the hospital environment, an obvious hub for patient colonization, prompted us to ask how environmental strains are related to patient-colonizing strains. To examine this, we constructed phylogenetic trees for environmental strains and patient isolates across species (Fig. 5). We started with Singaporean *E. anophelis* isolates from a 2012 outbreak^49^ (*n* = 10) and an additional set of patient isolates from 2009–2012 (*n* = 52; Fig. 5a and Methods). Despite sampling from different Singaporean hospitals after a span of 5–8 years, patient-associated genomes matched environmental genomes with just 16 SNPs (s1; 99.9996% ANI). The environmental *E. anophelis* genomes in our studies primarily originated from sinks, which, as noted earlier, tend to have stable communities, indicating that these strains may have originated from a common reservoir upstream of water-piping systems^39^. The *E. anophelis* clusters shared between patients and the environment were also detected at the third time point 1.5 years later (>99.999% ANI, direct transfer) and exhibited resistance to more antibiotics than the clusters that were not shared (1.25-foldchange; one-sided Wilcoxon *P* value = 0.059).

We next analyzed *S. aureus* genomes (*n* = 221) from a surveillance study of patients in the same hospital almost a decade ago^50^. These strains matched 5 of 17 strains obtained in the current study, with environmental and patient genomes having just 39 SNPs and 99.9985% ANI (Fig. 5b). The co-occurrence of patient and environmental genomes was significantly enriched in multidrug-resistant clusters at the derivative genomes threshold (for example, s1, s2 and s3; binomial test *P* value < 10^−15^). These clusters were also enriched for genomes detected in the third time point (binomial test *P* value < 2 × 10^−7^) with <60 SNPs (99.998% ANI) from genomes in early time points, highlighting the stability of antibiotic-resistant derivative clusters in the hospital environment.

To extend these observations, *A. baumannii* patient isolates (*n* = 108) from a hospital surveillance cohort in Singapore established >8 years ago were sequenced. Many isolate genomes from this cohort had high identity to our environmental genomes (s6, 99.995% ANI) while being temporally separated by almost a decade. In addition, patient isolates that overlapped with environmental genomes were enriched for multidrug resistance (derivative clusters; binomial test *P* value < 4 × 10^−3^). Extending to a regional context, analysis of *A. baumannii* patient isolates (*n* = 36) from two major Kuwaiti hospitals^51^ with our environmental genomes (Fig. 5c) identified a shared derivative cluster resistant to all five antibiotics, including Singaporean and Kuwaiti patient isolate genomes at high identity (s6, >99.99% ANI). This highlights the presence of multidrug-resistant *A. baumannii* derivative clusters in hospital environments that are persistent, enriched in overlap with patient isolates and globally disseminated.

Similar patterns were observed recently for *S. epidermidis* lineages (ST2/ST2 mixed), which seem to have disseminated globally within a short period of time (*n* = 229; ref. ^29^). We confirmed detection of these rifampicin-resistant^29^ lineages in our data (Fig. 5d), with 80 SNPs (99.997% ANI) from our hospital-environment genomes. One other lineage (ST16) not known to be globally disseminated (isolated from a patient sample in the United States^29^) was represented by a genome in our database with similarity at the derivative threshold (99.991% ANI). Finally, we found that the overlap between *S. epidermidis* patient isolates (surveillance samples from Austin Health in Australia^29^) and environmental genomes from this study was enriched for multidrug resistance in derivative clusters (binomial test *P* value < 4 × 10^−12^). Together with the observation that multiantibiotic-resistant strains are persistent and widely distributed across the hospital environment (Fig. 3d and Supplementary Fig. 11), these data point to selective advantages for MDROs to persist and spread in hospital environments and patients.

## Discussion

While the importance of hospital design for preventing infections is known^52^, the utility of metagenomic surveys in medical facilities remains underexplored^12^. A detailed survey helps provide a reference map (with three-dimensional (3D) visualization; https://github.com/csb5/hospital_microbiome_explorer) that can be updated based on periodic scans whose frequency and locations can be informed by the initial survey. For example, the turnover score and specificity of a site can determine whether and how frequently it should be sampled. Variations in human influence scores could fine-tune cleaning practices, and distribution of specific pathogens could inform infection control in outbreak settings. As genomics-guided infection control advances, this knowledge could feed back into better hospital designs. With further improvements in the cost and ease of short-read sequencing, hospital-wide surveys will be increasingly feasible, provide valuable information for infection control and eventually be part of routine practice.

The microbial community types observed here highlight distinct niches found in hospitals compared to other urban environments, providing an organizing principle for further study. For example, while many pathogens were substantially enriched in hospitals, this was also prominent in CTA sites that had a greater diversity of ARGs (Supplementary Fig. 7c,d). Recent clinical studies have focused on wash-area sites (such as sinks and showers; CTB sites), as outbreak-associated pathogens are often isolated there^39^. This focus on CTB sites is concordant with the presence of biofilm-forming bacteria and their harboring viable reservoirs for extended periods (for example, in the plumbing). Our data show that many pathogens (for example, *K. pneumoniae*), ARGs (for example, carbapenemases such as *oxa-23*) and ARG-containing plasmids (in >85% of sites) are more common in CTA sites. While CTA sites have higher turnover, the detection of highly similar strains over extended periods indicates that they have distinct reservoirs (for example, in ventilation or air-conditioning ducts) and that culture-based screening may bias against sites with lower biomass or variable colonization. Combining the strengths of metagenomics and culturing may therefore be needed to systematically explore the source of outbreaks.

Large-scale genomics of nosocomial pathogens through isolation can be laborious and time consuming, while metagenomics may not provide genomes for low abundance species. The intermediate approach proposed here addresses both issues. Culture-based enrichment allows us to shift the distribution away from abundant species (for example, *C. acnes*) and toward pathogens at low abundances (for example, *K. pneumoniae*, *S. aureus* and *A. baumannii*) while allowing functional selection such as for antibiotic resistance. Culture-based enrichment in combination with long-read metagenomics is powerful, enabling direct recovery of genomes (chromosomal, plasmid and phage) without isolation. With further automation (for example, library preparation), this workflow can enable high-throughput analysis and wider surveillance, to achieve the vision of precision epidemiology for infectious diseases^53^. Future improvements in nanopore sequencing throughput and lower DNA-input requirements could accelerate time-to-answer via point-of-care usage and reduce or eliminate the culturing period.

The availability of many high-contiguity assemblies (>8 Gb; 2,347 genomes and 1,693 phage and 5,910 plasmid sequences) provides a unique resource for studying the distribution of strains and diversity of ARG cassettes in the hospital environment. Leveraging this, we observed that multidrug-resistant strains are preferentially distributed and persistent in hospitals across a range of species. This represents a worrisome pattern, with several explanations that warrant investigation. One scenario is that hospitals are repeatedly seeded by resistant strains that preferentially persist in the community (humans or environment). This explanation seems less plausible as some species where this pattern is observed are rarely found in humans (for example, *E. anophelis* and *A. baumannii*), and it is based on observations that other urban microbiomes are distinct from hospitals in taxonomic composition, the frequency at which they harbor pathogens and diversity of ARGs. Nevertheless, this does not rule out the possibility that urban environments (1) harbor pathogens and resistant strains at lower abundances compared to hospitals and (2) resistant strains are also widespread and persistent in these environments. Another hypothesis is that hospital cleaning measures select for more antibiotic-resistant organisms^54^, a model that is supported by the presence of multiple copies of disinfectant resistance genes in widely distributed multidrug-resistant *S. aureus* strains in our study. Comparisons with surveys from built environments that are intensively cleaned but do not house patients (for example, operating rooms) or are not intensively cleaned but have high patient traffic (for example, clinic waiting areas) can help explore this hypothesis. Studies across wards and in hospitals with different protocols could also reveal how ARG reservoirs are shaped by cleaning practices^55^.

Despite their importance as an epicenter for the battle against growing antibiotic resistance^1^, hospital environments have received little attention compared to agricultural and animal farms^12^. Our analysis highlights that hospitals harbor a significant uncharacterized diversity of microbes (*n* = 13 novel species) and ARG combinations (*n* = 255). This reservoir can be the origin of new opportunistic infections or fertile ground for the evolution of clinically relevant ARG combinations (for example, colistin and fosfomycin resistance). In particular, the prevalence of plasmids containing ARGs (*n* = 1,400) could enable gene transfer across species^56^. The development and use of anti-plasmid agents^57^ could thus be a complementary strategy to curb the spread of ARGs through hospital environments.

While most studies have focused on patient isolates^58^, relatedness between environmental and patient-colonizing strains is important for understanding the risk that environmental strains pose^15,17^. For contemporary and co-located strains, high relatedness between a subset is expected. Despite samples being separated by >8 years, obtaining highly similar genomes suggests that large reservoirs of multidrug-resistant strains are maintained with limited diversification. The identification and elimination of these reservoirs may reduce the incidence of corresponding infections and the risk from maintenance of ARGs. Another interesting observation is the high genomic similarity between MDROs in Singaporean hospitals and those from patients globally. The consistency of these patterns across species emphasizes the global dissemination of newly emerging MDRO lineages; thus, the role of hospital environments deserves investigation, leveraging multinational metagenomic datasets^26^.

Overall, our data indicate selective advantages for MDROs to persist and spread in hospital environments (Fig. 3d and Supplementary Fig. 11) and be shared with patients (Fig. 5). The *S. aureus* derivative clusters that persisted in the hospital are enriched in virulence factors (1.5-fold; one-sided Wilcoxon *P* value = 0.015) and have three copies of disinfectant resistance genes^38,59^ (Fig. 3d), potentially enabling colonization of hospital environments and patients and facilitating transfer between them. This points to a vicious cycle where disinfectant resistance, antibiotic resistance and virulence may in turn be selected for, enriching for strains adept at colonizing both niches with depleted microbial competition and offering an explanation for the high incidence of multidrug-resistant HAIs worldwide despite increased surveillance and aggressive cleaning measures in hospitals^60^.

## Methods

### Sample collection and storage

Environmental swabs were collected from Tan Tock Seng Hospital (TTSH), a major tertiary-care hospital with >2,000 patient visits daily, serving as the national referral center for communicable diseases in Singapore. Sampling was conducted in November 2017 and in May 2019. Samples were collected in 2 days for the first time point and in 3 days for the second time point, with 1 week separating the time points. The third time point was 1.5 years later, with samples collected in 4 days across 2 weeks. Samples were collected from isolation rooms (1 bed, typically for patients colonized with CRE), MDRO wards (5 beds, typically for patients colonized with MRSA) and standard wards (5 beds) at seven different sites, including the aerator, sink trap, bed rail, bedside locker, cardiac table, pulse oximeter and door handle (Fig. 1 and Supplementary Data 1). Standard cleaning protocols at TTSH require that high-touch areas and sinks be cleaned daily with chlorine (5,000 ppm) and cleaning detergent, respectively, excluding beds that are cleaned upon patient discharge. Isohelix DNA Buccal Swabs (SK-4S) were used for sampling according to MetaSUB protocols^26^. Briefly, a total of four swabs were collected; one swab (for culturing) was moistened with 1× PBS (pH 7.2), and three swabs (two swabs for metagenomic DNA isolation and one swab for storage) were moistened with DNA/RNA shield (Zymo Research, ZYR.R1100-250). Swabbing was performed for 2 min in each site, and swabs were stored in respective storage liquids (that is, 1× PBS, pH 7.2, or Zymo DNA/RNA shield). Swabs in PBS were placed on ice and sent for culturing while the other swabs were transported at room temperature to the laboratory and stored at −80 °C. In total, 1,752 swabs were collected from 179 sites in the hospital at three time points, representing 438 unique samples. Swabs were also collected from an office environment (Genome Institute of Singapore) with sites selected to approximately match those from which samples were collected in the hospital (aerator, sink trap, chair handle, office desk, keyboard and door handle; *n* = 30; Supplementary Data 1). MetaSUB Singapore samples were collected from high-touch surfaces in different parts of the city and analyzed based on MetaSUB protocols as described in Danko et al.^26^ (*n* = 99; Supplementary Data 1).

### DNA extraction from swabs

DNA was extracted from swabs using a bead-beating and automated DNA purification system. Briefly, 300 µl of lysis buffer was added to Lysing Matrix E tubes (MP Biomedicals, 116914500). Samples were homogenized using the FastPrep-24 instrument at 6 m s^–1^ for 40 s before centrifugation at maximum speed for 5 min. The supernatant was treated with proteinase K (Qiagen Singapore, 19133) for 20 min at 56 °C before DNA was purified with the Maxwell RSC Blood DNA Kit (Promega, AS1400). DNA concentration was quantified using a Qubit 2.0 fluorometer, prepared with the Qubit dsDNA HS Assay Kit (Life Technologies, Q32854). DNA extraction from backup swabs was carried out for samples with insufficient amounts of DNA. Samples that still had less than 0.5 ng of DNA were excluded from library preparation (10 of 438).

### Illumina library preparation

Extracted DNA was sheared using Adaptive Focused Acoustics (Covaris) with the following parameters: 240 s, duty factor of 30, PIP of 450 and 200 cycles per burst. Metagenomic libraries for the first two time points were prepared with the NEBNext Ultra DNA Kit (New England Biolabs, E7370) according to the manufacturer’s instructions. Paired-end sequencing (2 × 101-bp reads) was performed on the Illumina HiSeq2500 platform. For the third time point, metagenomic libraries were prepared using the NEBNext Ultra II DNA Library Prep Kit (New England Biolabs, E7645) according to the manufacturer’s instructions. Paired-end sequencing (2 × 151-bp reads) was performed on the Illumina HiSeq 4000 platform.

### Culture enrichment

Following MetaSUB protocols, swabs were directly incubated with 7 ml of Brain Heart Infusion (BHI) broth (Thermo Scientific Microbiology, CM1135B) at 37 °C until turbidity was observed (14–16 h for >95% of samples), up to a maximum of 48 h. Culture tubes were centrifuged at 3,200*g* for 12 min. For the first two time points, cell pellets were resuspended with 550 µl of 1× PBS, while the cell pellets for the third time point were resuspended with 1 ml of 1× PBS. Fifty microliters of resuspended cultures was then plated on each of six agar plates (without antibiotics, BHI; ampicillin: 100 µg ml^–1^, AMP; streptomycin sulfate: 100 µg ml^–1^, STREP; tetracycline: 10 µg ml^–1^, TET; kanamycin: 50 µg ml^–1^, KAN; and chloramphenicol: 35 µg ml^–1^, CHLOR), and plates were incubated overnight at 37 °C. Cells were harvested by a plate sweep and were pelleted by centrifugation at 8,000*g* for 15 min at 4 °C for the first two time points. For the third time point, a loopful of harvested cells was streaked out on an antibiotic-free BHI plate to obtain single colonies for whole-genome sequencing. Plates were only excluded if no cells were growing on the plates or when the growth was insufficient to generate enough DNA for sequencing.

### DNA extraction from enrichment cultures

Frozen cells were thawed on ice and manually mixed with a wide-bore pipette tip. A volume of 30–50 µl of cells was resuspended in 100 µl of 1× PBS (pH 7.4). Twenty microliters of suspended cells was added to 20 µl of metapolyzyme (6.7 µg µl^–1^; Sigma Aldrich, MAC4L). The mixture was incubated at 35 °C for 4 h. RNase treatment was carried out by adding 350 µl of 1× TE buffer and 10 µl of RNase A (4 mg µl^–1^) and incubating on a rotator for 10 min at room temperature. DNA was extracted with the Maxwell RSC Cultured Cells Kit (Promega, AS1620). DNA was cleaned up and concentrated with 0.4× Agencourt AMPure XP beads (Beckman Coulter, A63882). DNA purity and concentration were measured with a NanoDrop and Qubit fluorometer. DNA integrity was assessed on a 0.5% agarose gel. DNA samples with the following quality measurements were selected for nanopore sequencing: DNA amount: >400 ng; A260/280: 1.8–2.0; A260/230: 1.7–3.0; Qubit:NanoDrop: 0.7–1.3; DNA integrity on 0.5% agarose gel: >1 kb. The Qubit:NanoDrop ratio was used to estimate and control the amount of single-stranded DNA in the sample and ensure successful nanopore sequencing.

### Collection and testing of bacterial isolates from patients

*E. anophelis* isolates (*n* = 52) were obtained from consecutive positive blood cultures and respiratory samples collected in a 3-year period (2009–2012) at the National University Hospital in Singapore (DSRB reference 2017/00879). *A. baumannii* complex isolates (*n* = 108) were consecutively obtained from all clinical specimens (including blood, tissue, respiratory and urine samples) sent for routine bacterial culture between February 2009 and May 2009 at the Singapore General Hospital Diagnostic Bacteriology Laboratory (de-identified and archived, hence institutional review board approval was not required). Antibiotic susceptibility testing for *E. anophelis* isolates was performed with 13 antimicrobial agents (cefotaxime, ceftazadime, cefepime, imipenem, meropenem, ampicillin-sulbactam, piperacillin/tazobactam, tigecycline, gentamicin, nalidixic acid, ciprofloxacin, levofloxacin and trimethoprim/sulfamethoxazole) using Etest strips (bioMérieux). Minimum inhibitory concentrations (MICs) were interpreted according to the Clinical and Laboratory Standards Institute (CLSI) guidelines for non-Enterobacteriaceae Gram-negative bacilli (performance standards for antimicrobial susceptibility testing, M100-S22 and CLSI 2012; Supplementary Data 6). Antibiotics to which all strains were resistant were excluded from statistical analysis. Antibiotic susceptibility testing for *A. baumannii* complex isolates was conducted with 11 antimicrobial agents (ampicillin/sulbactam, piperacillin/tazobactam, cefepime, imipenem, gentamicin, amikacin, ciprofloxacin, levofloxacin, trimethoprim/sulfamethoxazole, minocycline and polymixin B). Polymixin B susceptibility testing was performed using Etest strips (bioMérieux), and disk diffusion was performed for all other antimicrobial agents. Polymixin B MICs and zone diameters for all other tested agents were interpreted in accordance with CLSI breakpoints for *Acinetobacter* spp. (performance standards for antimicrobial susceptibility testing, M100-S19 and CLSI 2009; Supplementary Data 6). Multidrug-resistant status for patient isolates were defined according to US Centers for Disease Control and Prevention (CDC) guidelines (https://www.cdc.gov/nhsn/pdfs/ps-analysis-resources/phenotype_definitions.pdf).

### DNA extraction for bacterial isolates

Cell pellets were allowed to thaw slowly on ice and resuspended in 400 µl of ATL buffer (Qiagen Singapore, 19076). Cells were lysed in Lysing Matrix E tubes (MP Biomedicals, 116914500) on a vortex adapter at maximum speed for 10 min. Cell lysates were centrifuged at 16,000*g* for 5 min, and supernatant was treated with 4 µl of RNase A (100 mg ml^–1^; Qiagen Singapore, 19101), gently mixed by flicking of the tube and incubated at room temperature for 2 min. The cell lysate was further treated with 25 µl of proteinase K (20 mg ml^–1^; Qiagen Singapore, 19133), gently mixed by flicking of the tube and incubated at 56 °C for 20 min. DNA was purified twice using 1 volume of AMPure XP beads (Beckman Coulter, A63882) with slight modifications to the manufacturer’s protocol. All mixing steps were replaced with gentle flicking of the tube and incubation on the hula rotor for gentle mixing. Fresh 70% ethanol was prepared for washing, and magnetic beads were incubated on a 37 °C heat block for 3–5 min to dry off residual ethanol. The quality and quantity of DNA were assessed using a NanoDrop, Qubit fluorometer and 0.5% agarose gel. Samples that were unable to pass the following criteria were omitted from sequencing: DNA amount measured by Qubit: >510 ng; DNA concentration measured by Qubit: >11 ng µl^–1^. A260/280 ratio: between 1.7–2.0; A260/230 ratio: between 1.5–3.3; and DNA length: >1 kb. Purified DNA was stored at 4 °C.

### Nanopore library preparation

DNA was prepared with either the 1D^2^ sequencing kit (SQK-LSK308) or the 1D sequencing kit (SQK-LSK108 or SQK-LSK109) together with the native barcoding kit (EXP-NBD103 or EXP-NBD104 and EXP-NBD114) according to the native barcoding genomic DNA protocol. DNA was not sheared and was used directly for DNA repair and end preparation. Both native barcode ligation and adaptor ligation steps were extended to 30 min instead of 10 min. In addition, to maximize library yields, more than 700 ng of pooled sample (where possible) was used for adaptor ligation. Samples were multiplexed (9–12 samples for each pool for culture-enriched samples and 24 samples for each pool for isolates) and sequenced with MIN106, MIN106D or MIN107 flowcells on a GridION machine.

### Taxonomic and resistome profiling with Illumina shotgun metagenomic data

Illumina shotgun metagenomic sequencing reads were processed using a Snakemake pipeline (https://github.com/gis-rpd/pipelines/tree/master/metagenomics/shotgun-metagenomics). Briefly, raw reads were filtered to remove low-quality bases using skewer (v0.2.2; -q 3 -l 30 -n) and human reads were removed by mapping to the hg19 reference using BWA-MEM (v0.7.10-r789). The remaining microbial reads were profiled with MetaPhlAn2 (ref. ^61^; v2.6.0) and SRST2 (ref. ^62^; v0.1.4; --min_coverage 100, hits with identity <99% were filtered out) for taxonomic and ARG abundances, respectively. Microbial reads were also assembled using MEGAHIT (v1.0.4-beta; default parameters) for comparison to nanopore assemblies. The site specificity score was computed as the *z*-score for the closest taxonomic profile for a sample (Bray–Curtis dissimilarity) among physically proximal sites (in the same room or cubicle and at the same time point), compared to the distribution of Bray–Curtis dissimilarities across all samples of a site (for example, all bed rails). Results based on analysis of taxonomic and resistome profiles were obtained for each time point independently and compared across time points to check for consistency and filter out potential sequencing artefacts^23^.

### Removal of likely contaminant species

Likely contaminant species were identified based on batch and correlation analysis^23^ (Supplementary Note 2) and were removed from species-level abundance profiles. For genus-level profiles, relative abundances of the filtered species were subtracted from the abundance of the corresponding genera for each sample. Filtered profiles were then renormalized to sum to 100% and used for all downstream analyses.

### Preprocessing of nanopore sequencing data

Raw nanopore reads were base-called with the latest version of the basecaller available at the point of sequencing (Guppy v0.5.1 to v3.0.6 or Albacore v2.3.1 to v2.3.3, for libraries that failed live base-calling). Base-called nanopore reads were demultiplexed and filtered for adaptors with Porechop (v0.2.3; https://github.com/rrwick/Porechop) or qcat (v.1.1.0; https://github.com/nanoporetech/qcat). Sequencing statistics were summarized using SeqKit (v0.10.1). Reads were taxonomically classified with Kraken^63^ (v0.10.5-beta) against the miniKraken database to assess the diversity of cultures on the plates (minikraken_201711_01_8GB_dustmasked).

### Genome assembly and species assignment

Nanopore reads for each plate were assembled using Canu^64^ (v1.3 and v1.7; genomeSize = 8 m). For samples where both Illumina and nanopore reads were available, a higher-quality hybrid assembly was obtained using OPERA-MS^19^ (v0.8.3; --polish --no-gap-filling --short-read-assembler spades). Assembled contigs were mapped to the NCBI nt database with BLAST (v2.2.28), to identify microbial species or plasmid assignments according to the best BLAST hit (highest total reference coverage). Circular sequences were identified using MUMmer^65^ (v3.23; --maxmatch --nosimplify, alignments <1 kb long or with identity <95% were filtered out) as recommended in the documentation for Canu (https://canu.readthedocs.io/en/latest/faq.html#my-circular-element-is-duplicated-has-overlap). Contigs assigned to the same species were binned into genomic bins. Metagenomic Illumina reads were used to polish Canu assemblies where feasible using Pilon^66^ (v1.22; --fix indel). We noted that annotation errors were substantially reduced after polishing and that genomic bins whose length was within 10% of the expected length met the criteria for high-quality genomes (completeness > 90% and contamination < 5% using CheckM^67^; v1.0.7; --reduced_tree). Genomic bins that met these criteria were therefore designated as high quality, and incomplete bins (<50% of the expected length) were removed from further analysis. Genomes corresponding to novel species were identified as those with identity <95% or coverage <80% when compared with known genomes (BLAST with nt) and three recent catalogs that include environmental and human microbiome assembled genomes^31–33^ (with Mash^68^). The genomes were hierarchically clustered (single linkage with Mash distance^68^) to identify species-level clusters at 95% identity, and genus-level taxonomic classification was obtained using sourmash^69^. Similarly, novel circular plasmids were identified by comparing to the PLSDB^36^ database with Mash distance and identifying clusters at 99% identity (single linkage) with no known sequence.

### Analysis of ARG combinations

ARGs were annotated to contigs by mapping them to the ARG-ANNOT^70^ database provided in SRST2 (v3) with BLAST (best hit with >90% identity and >90% reference coverage). ARG combinations present in chromosomes and plasmid sequences were considered novel when they were not found in the reference databases (nt or PLSDB^36^). Assembled circular plasmids were clustered and annotated based on their best BLAST hit with identity >95% and >60% query coverage. A bipartite graph was constructed by connecting each plasmid cluster to ARGs found in it, with edge weights representing the frequency of occurrence (clusters with <5 representatives were excluded). For each species, an ARG co-occurrence graph was created for ARGs found in the assembled genomes by connecting the ARG pairs that were found within 10 kb on the same contig (discarding ARG pairs occurring fewer than five times). Each edge was weighted by the frequency of ARG pairs divided by the minimal frequency of the two ARGs. All ARG co-occurrence graphs were merged into a final co-occurrence multigraph. The graphs were visualized using Cytoscape (v3.7.1).

### Analysis of virulence factor and biocide resistance genes

Nanopore assemblies were aligned to virulence factors in the PATRIC database^71^ (20 December 2018) with DIAMOND (v0.9.24.125; blastx --long-reads), and alignments with *E* value > 0.001 were filtered out. To identify biocide resistance genes, the assemblies were aligned to nucleotide sequences for the genes *qacA* (NC_014369.1) and *qacC* (NC_013339.1) with BLAST (>90% identity and >90% reference coverage).

### Analysis of phages and prophages

Phage-like elements (phages and prophages) were identified using VirSorter^72^ (v1.0.5; phages and prophages in category 3 or with length <10 kb were filtered out). The assembled phages and reference phages from the MVP database^37^ were hierarchically clustered (single linkage with Mash distance^68^) to identify phage clusters at 95% identity. Clusters without any phages from the reference database were considered novel. For each cluster, subclusters were defined at 99.9% ANI by single-linkage clustering with nucleotide identities from nucmer (--maxmatch --nosimplify, followed by dnadiff and minimum sequence overlap of 80%). Phage-like elements were annotated using RAST^73^ (virus domain, fix frame-shifts parameters).

### Analysis of patient isolates and strain relationships

Raw reads corresponding to genomes for outbreak isolates^29,49–51^ were downloaded and assembled using the Velvet assembler (v1.2.10) with parameters optimized by Velvet Optimiser (*k*-mer length: ranging from 81–127), scaffolded with OPERA-LG^74^ (v1.4.1) and gap-filled with FinIS^75^ (v0.3). Outbreak genomes from the same species were jointly analyzed with high-quality genomes from the hospital environment. To identify high-confidence SNPs, we adapted the method from Brooks et al.^17^. Specifically, we performed pairwise alignments between genomes using nucmer and considered genome pairs with alignment coverage > 80% for ANI computation. SNPs between genome pairs were called using MUMmer’s ‘show-snps’ function, and regions containing more than one SNP within 20 bp were filtered out to mask potential artefacts from horizontal gene transfer, recombination or repeats. Finally, the genomic distance matrix (number of SNPs/alignment size) was clustered hierarchically (single linkage) and clusters were obtained at 99.99% identity for species with hybrid assemblies (nanopore and Illumina) or at 99.9% identity for species with nanopore-only assemblies. Single-linkage clustering was used to avoid having highly similar genomes assigned to separate clusters, and we confirmed that despite this, most members (99%) had an average distance to other members of the cluster below the clustering thresholds used. Antibiotic resistance profiles and multidrug resistance status (>2 antibiotic types) for each cluster were derived from the union of resistance profiles for each genome obtained in various selection plates.

For phylogenetic analysis, a consensus genome was derived for each cluster based on reference-guided alignment with nucmer (*S. aureus*: NC_020529; *S. epidermidis*: NC_004461; *E. anophelis*: NZ_CP007547; *E. faecalis*: NC_017312; *E. faecium*: NC_017960; *P. aeruginosa*: NC_018080; *K. pneumoniae*: NC_018522; and *A. baumannii*: NC_009085) and the cons utility in the EMBOSS suite. Maximum-likelihood phylogenetic trees were constructed for each species with Parsnp^76^ (v1.2; -c -x; accounting for recombination events using PhiPack^77^) based on consensus genomes for each cluster, where multiple-sequence alignments for each species varied in length from 0.6 Mb (*S. epidermidis*) to 5.1 Mb (*P. aeruginosa*). For the species-level tree, full-length 16S rRNA sequences (*S. epidermidis*: L37605.1; *S. aureus*: NR_118997.2; *E. anophelis*: NR_116021.1; and *A. baumannii*: NR_026206.1) were aligned with MAFFT (v7.154b; default parameters) and the phylogeny was determined using FastTree2 (ref. ^78^; v2.1.8; default parameters). The trees were visualized using the ‘ggtree’ R package^79^. Strain distributions across sites were visualized with the ‘HiveR’ R package (https://github.com/bryanhanson/HiveR). Rarefaction analysis for species, plasmids, strains and resistance genes was performed using the iNEXT R package^80^.

### Statistical analysis

Statistical tests were performed using R and were two sided unless otherwise specified. For enrichment analysis at the cluster level (overlap across time, cohorts or resistance status), Fisher’s exact test was used. The binomial test was used for analysis at the genome level (fraction of genomes with a specific property).

### Reporting Summary

Further information on research design is available in the Nature Research Reporting Summary linked to this article.

## Online content

Any methods, additional references, Nature Research reporting summaries, source data, extended data, supplementary information, acknowledgements, peer review information; details of author contributions and competing interests; and statements of data and code availability are available at 10.1038/s41591-020-0894-4.

## Supplementary information

Supplementary Information Supplementary Figs. 1–13 and Supplementary Notes 1–3

Reporting Summary

Supplementary Data 1 Metadata for sampled sites.

Supplementary Data 2 Taxonomic and resistome profiles.

Supplementary Data 3 Metadata for culture plates and isolates.

Supplementary Data 4 Novel antibiotic resistance gene combinations.

Supplementary Data 5 Common resistance gene cassettes.

Supplementary Data 6 Antibiograms for Singaporean patient isolates.

## Data Availability

Sequencing reads and assemblies are available from the European Nucleotide Archive under project PRJEB31632. Source code and data for reproducing figures are available under MIT license at https://github.com/csb5/hospital_microbiome. Assemblies and annotations for genomes, plasmids and phages are available at https://t.co/bdZxADGM7z.
