# Supplementary material for: Cartography of opportunistic pathogens and antibiotic resistance genes in a tertiary hospital environment
Source: Nat Med. 2020 Jun 8;26(6):941–51. doi: 10.1038/s41591-020-0894-4 (PMC7303012; doi:10.1038/s41591-020-0894-4)
Supplement: Supplementary file 1 — Supplementary Figs. 1–13 and Supplementary Notes 1–3 [file 41591_2020_894_MOESM1_ESM.pdf]

nature medicine

SUPPLEMENTARY INFORMATION

https://doi.org/10.1038/s41591-020-0894-4

In the format provided by the authors and unedited.

Cartography of opportunistic pathogens and antibiotic resistance genes in a tertiary hospital environment

Kern Rei Chng[1,60], Chenhao Li[1,60], Denis Bertrand[1,60], Amanda Hui Qi Ng[1], Junmei Samantha Kwah[1], Hwee Meng Low[1], Chengxuan Tong[1], Maanasa Natrajan[1], Michael Hongjie Zhang[1], Licheng Xu[2], Karrie Kwan Ki Ko[3,4,5], Eliza Xin Pei Ho[1], Tamar V. Av-Shalom[1], Jeanette Woon Pei Teo[6], Chiea Chuen Khor[1], MetaSUB Consortium*, Swaine L. Chen[1], Christopher E. Mason[7], Oon Tek Ng[8,9,10], Kalisvar Marimuthu[8,9,11], Brenda Ang[8,9] and Niranjan Nagarajan[1,11]✉

[1]Computational and Systems Biology, Genome Institute of Singapore, Singapore, Singapore. [2]Information Systems Technology and Design, Singapore University of Technology and Design, Singapore, Singapore. [3]Department of Microbiology, Singapore General Hospital, Singapore, Singapore. [4]Department of Molecular Pathology, Singapore General Hospital, Singapore, Singapore. [5]Duke-NUS Graduate Medical School, Singapore, Singapore. [6]Department of Laboratory Medicine, National University Hospital, Singapore, Singapore. [7]Department of Physiology and Biophysics, Weill Cornell Medicine, New York, NY, USA. [8]National Centre for Infectious Diseases, Singapore, Singapore. [9]Department of Infectious Diseases, Tan Tock Seng Hospital, Singapore, Singapore. [10]Lee Kong Chian School of Medicine, Nanyang Technological University, Singapore, Singapore. [11]Yong Loo Lin School of Medicine, National University of Singapore, Singapore, Singapore. [60]These authors contributed equally: Kern Rei Chng, Chenhao Li, Denis Bertrand. *A full list of authors and their affiliations appears at the end of the paper. ✉e-mail: nagarajann@gis.a-star.edu.sg

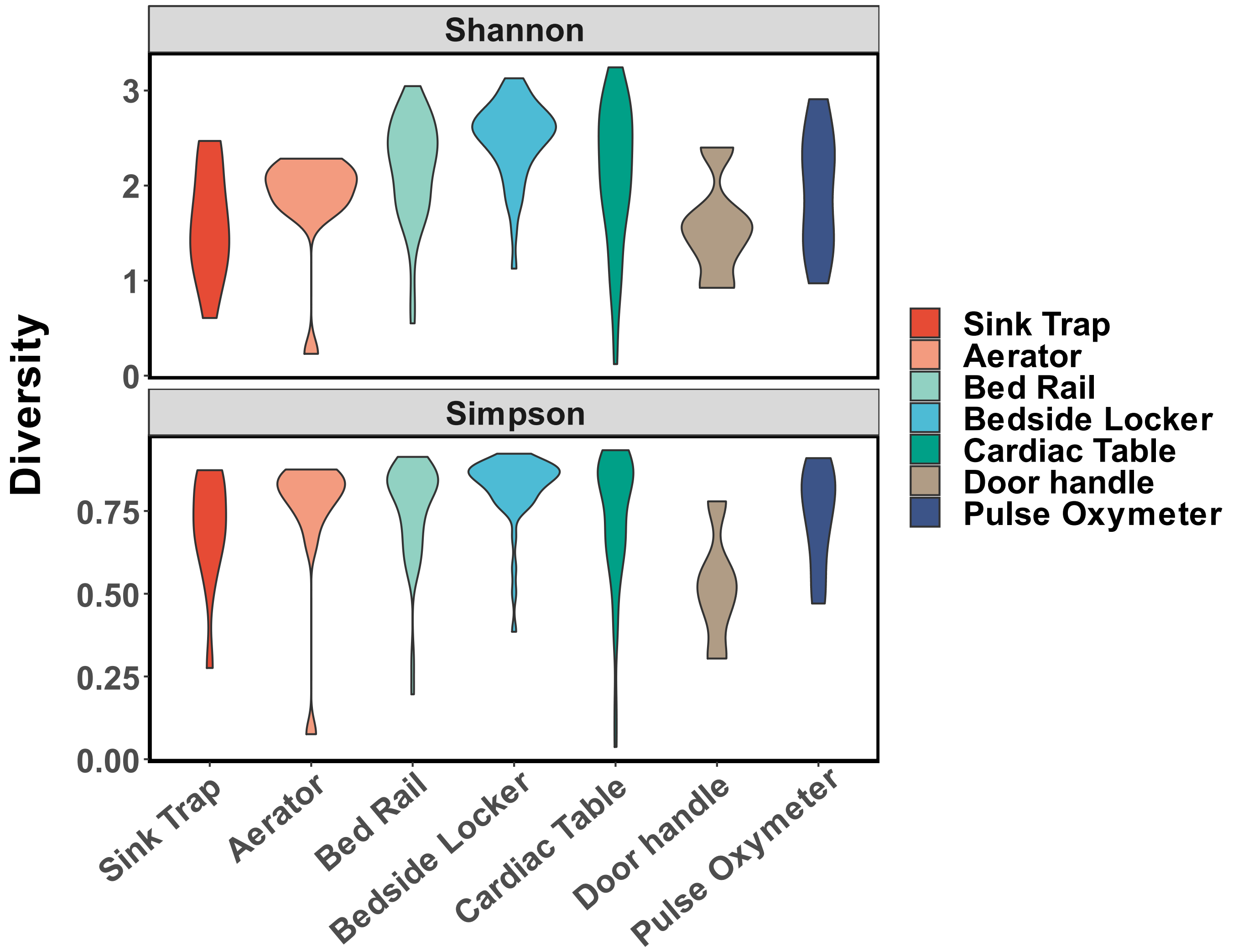

Supplementary Figure 1: Violin plots showing the distribution of genus-level (top) Shannon and (bottom) Simpson diversity metrics for different sampled sites (n=12, 13, 45, 45, 45, 5 and 11 for sink trap, aerator, bed rail bedside locker, cardiac table, door handle and pulse oxymeter, respectively). Shannon diversity of CTA sites was generally higher than CTB sites (two-sided Wilcoxon p-value<10^{-3}). The probability density of each violin plot was truncated at the minimum and maximum.

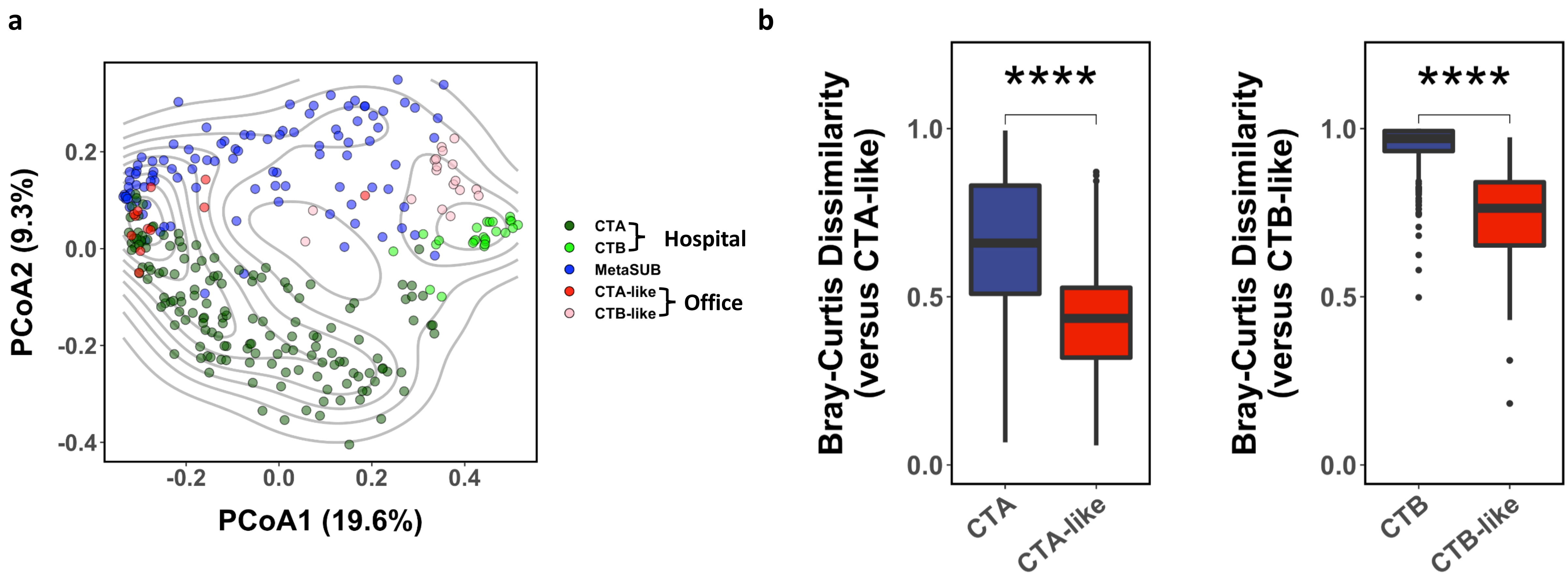

Supplementary Figure 2: a) Principle coordinates analysis plot (genus-level Bray-Curtis dissimilarity) based on taxonomic profiles for hospital (n=176 independent samples), office (n=30 independent samples) and other high-touch environmental microbiomes (MetaSUB, n=99 independent samples from Singapore). b) Boxplots showing that hospital CTA (n=1812 and 132 combinations for CTA and CTA-like, respectively) and CTB (n=450 and 306 combinations for CTB and CTB-like, respectively) microbiomes are distinct from corresponding office microbiomes (CTA-like: office desk, chair handle, door handle, keyboard; CTB-like: sink trap, aerator; genus-level Bray-Curtis dissimilarity, two-sided Wilcoxon p-value<10^{-15} for both tests). Boxplots are represented with center line: median; box limits: upper and lower quartiles; whiskers: 1.5× interquartile range; points: outliers 1.5× interquartile range away from the median. ****: p-value<0.0001.

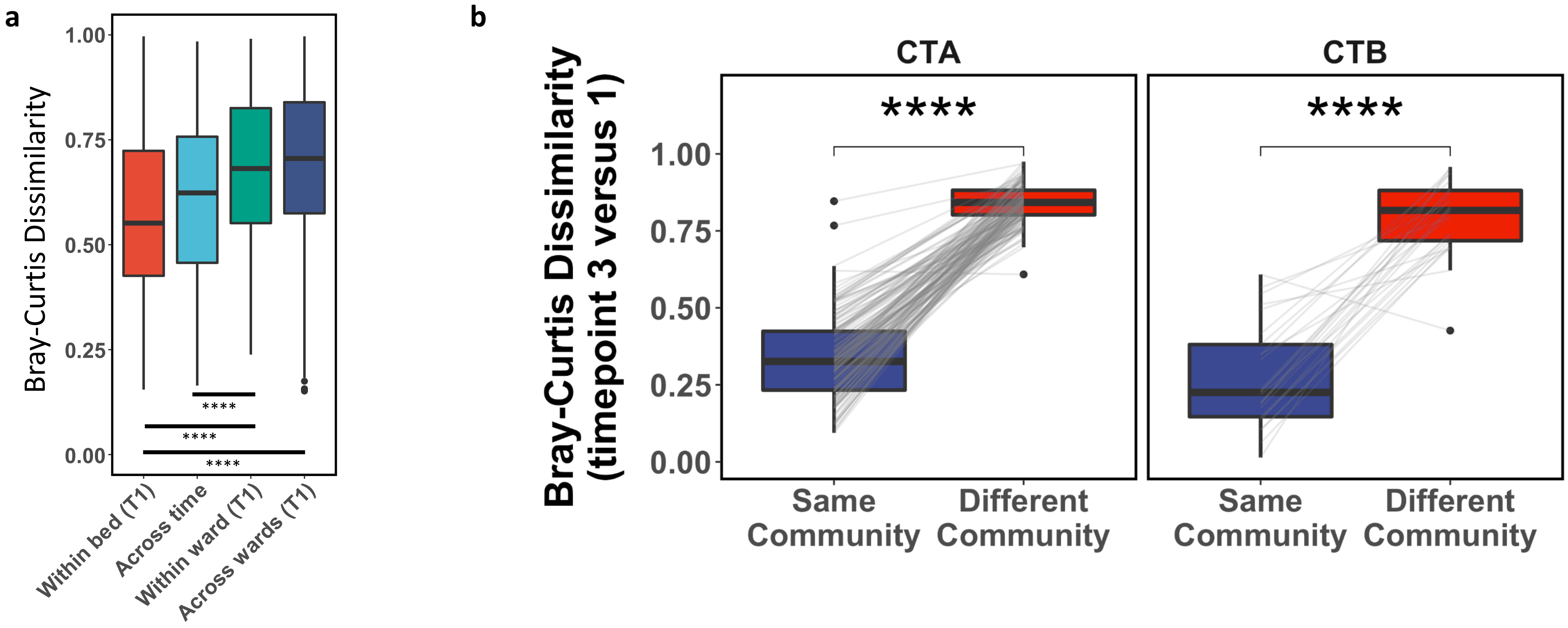

Supplementary Figure 3: a) Boxplots showing how dissimilarity between microbiomes (genus-level Bray-Curtis) varies as we move from sites surrounding the same bed (n=135 combinations) at one timepoint (cardiac table, bed rail and bedside locker; timepoint 1; n=270 combinations), to sites associated with the same bed across two timepoints one week apart, to sites in the same ward at one timepoint (timepoint 1; n=480 combinations), and finally to those in different wards (timepoint 1; n=5460 combinations). Dissimilarities in these sites increases significantly with physical distance (two-sided Wilcoxon p-value=4×10^{-7}, 5×10^{-11} and 2×10^{-6} for within bed vs. within ward, within bed vs. across wards and across time vs. within ward, respectively). b) Boxplots showing that community type identity is largely preserved from the 1st to 3rd timepoint (1.5 years apart), where community type A (left, n=151 pairs) or B (right, n=25 pairs) samples from the 3rd timepoint are much more similar to the same community type samples in the 1st timepoint (minimum genus-level Bray-Curtis dissimilarity, paired two-sided Wilcoxon p-value<1×10^{-15} and p-value=9×10^{-8} for CTA and CTB, respectively). Boxplots in a) and b) are represented with center line: median; box limits: upper and lower quartiles; whiskers: 1.5× interquartile range; points: outliers 1.5× interquartile range away from the median. ****: p-value<0.0001.

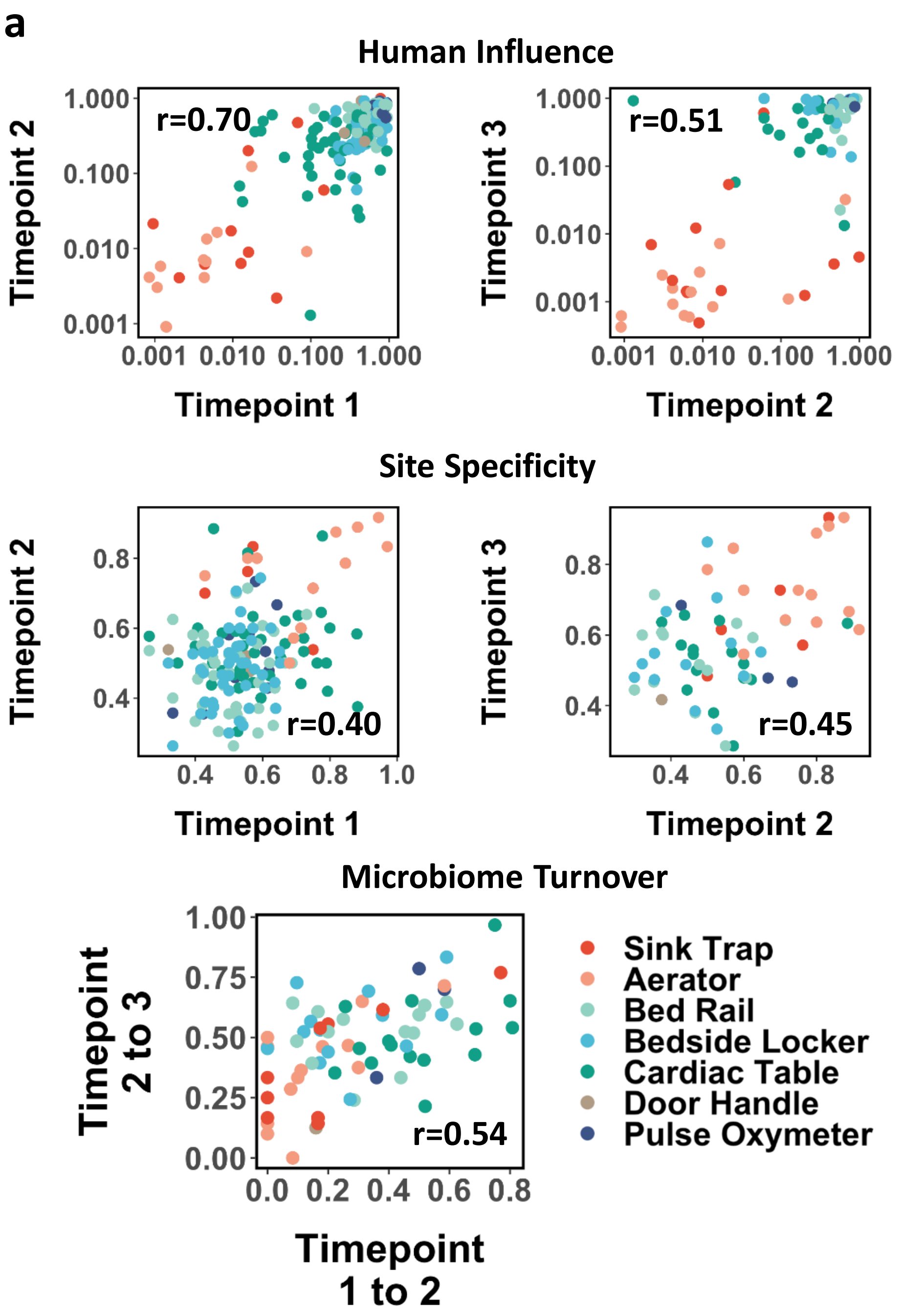

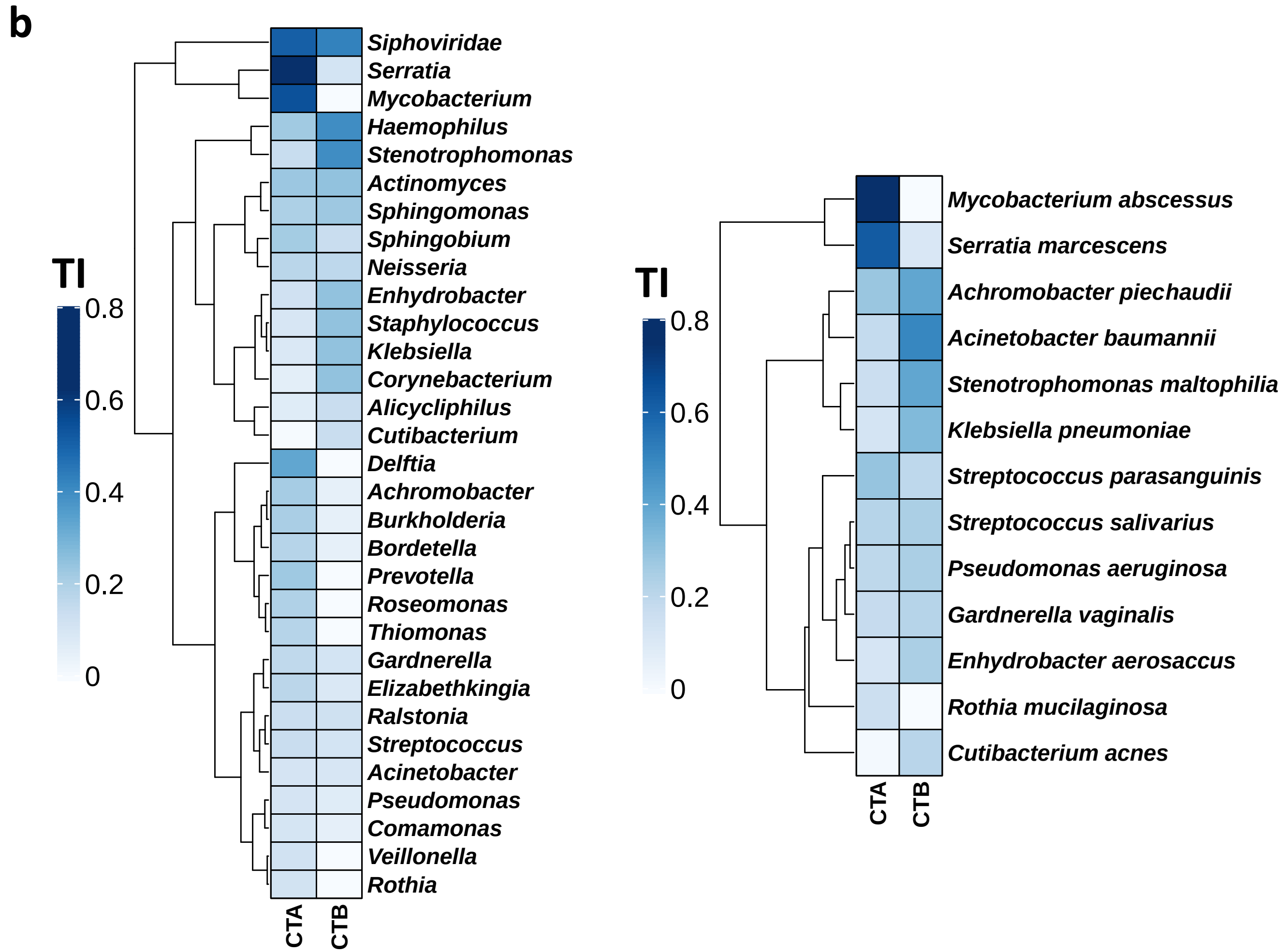

Supplementary Figure 4: a) Scatterplots showing significant pearson correlation between human influence (top panel; n=176 and 71 independent pairs for left and right figures, respectively; two-sided t-test p-value=3×10^{-8} and 5×10^{-5} for left and right figures, respectively), site specificity (middle panel; n=176 and 71 independent pairs for left and right figures, respectively; two-sided t-test p-value<10^{-15} and p-value=4×10^{-6} for left and right figures, respectively) and microbiome turnover (bottom panel; n=71 independent pairs; two-sided t-test p-value=9×10^{-7}) indices at various sites across time (up to 1.5 years apart). b) Heatmap representation of turnover index (TI i.e. fraction of sites where the taxa is gained or lost across timepoints 1 and 2) for (left panel) genus and (right panel) species that appear in both CTA and CTB sites. A low TI indicates that the genus/species is likely to persist at that specific site through time.

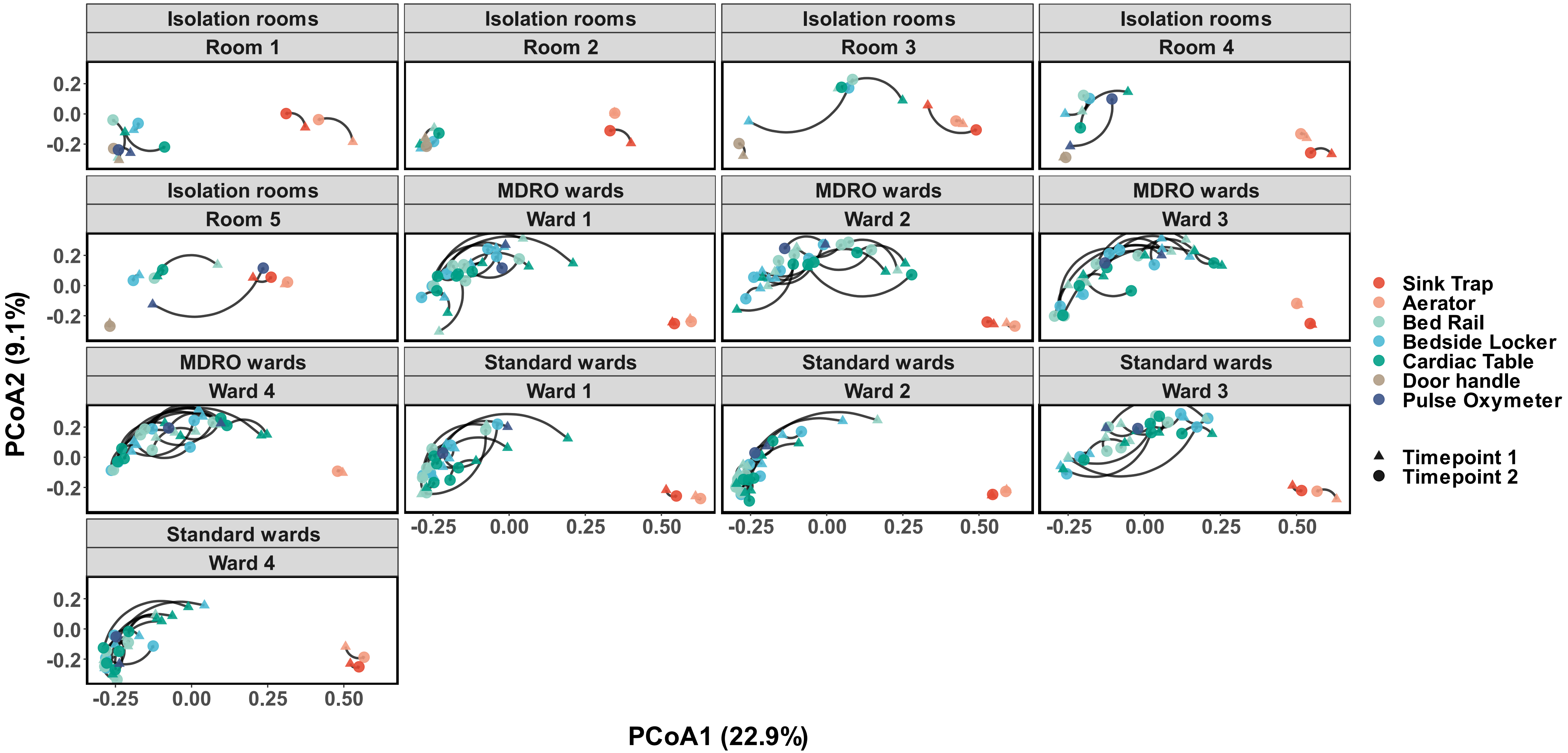

Supplementary Figure 5: Principle coordinates analysis (genus-level Bray-Curtis dissimilarity) of environmental microbiomes in different wards of the hospital (lines connect the same site across the two timepoints; n=14 for isolation room 1, 3, and 5, n=12 for isolation room 2 and 3, n=34 for MDRO ward 4 and n=36 for the others). Interestingly, CTB sites resemble CTA sites more in isolation rooms 3 and 5, while the figure further highlights the stability of CTB sites in general.

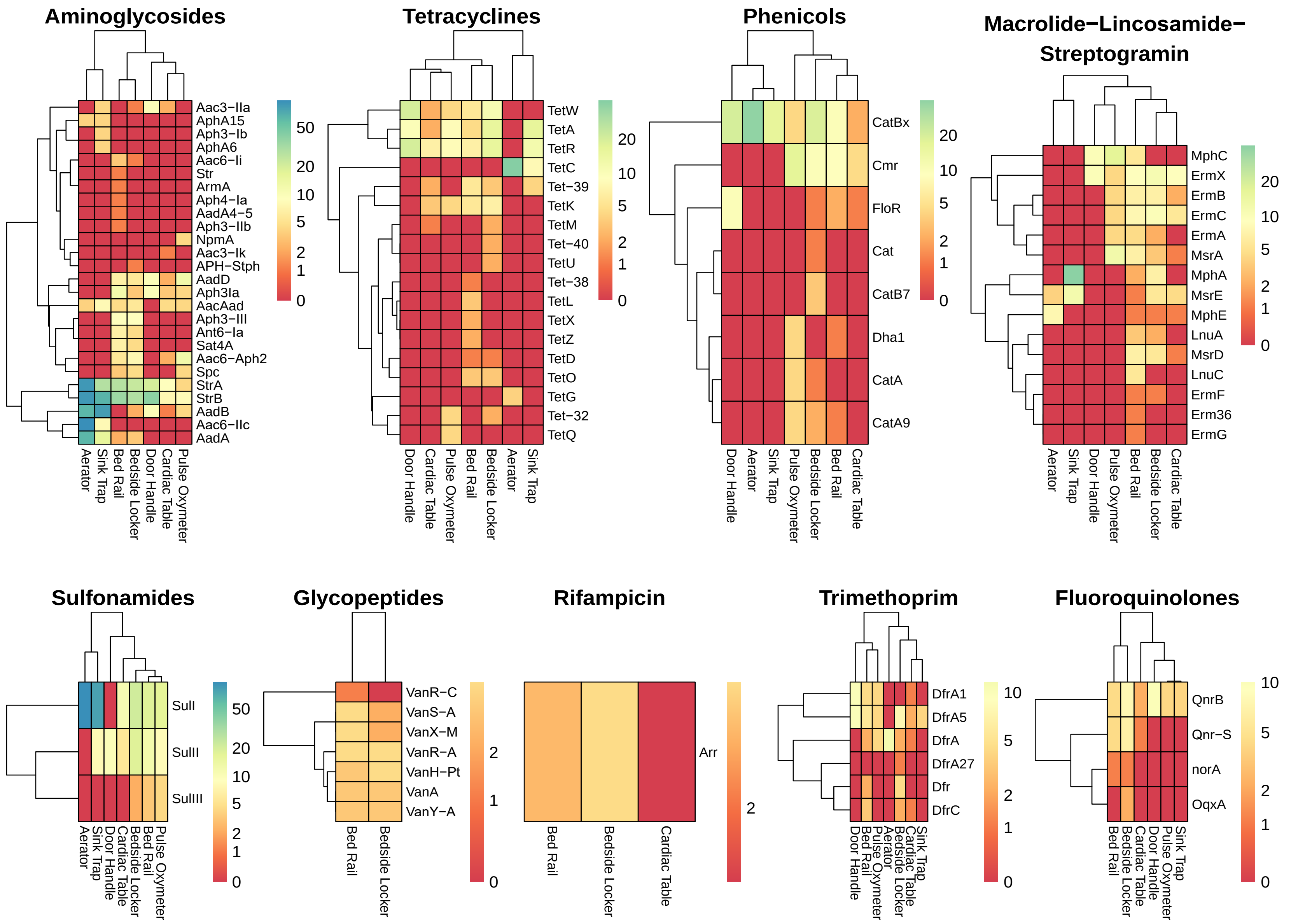

Supplementary Figure 6: Heatmaps showing the frequencies (percentage) at which antibiotic resistance genes were detected across sites. Zero values are depicted in red.

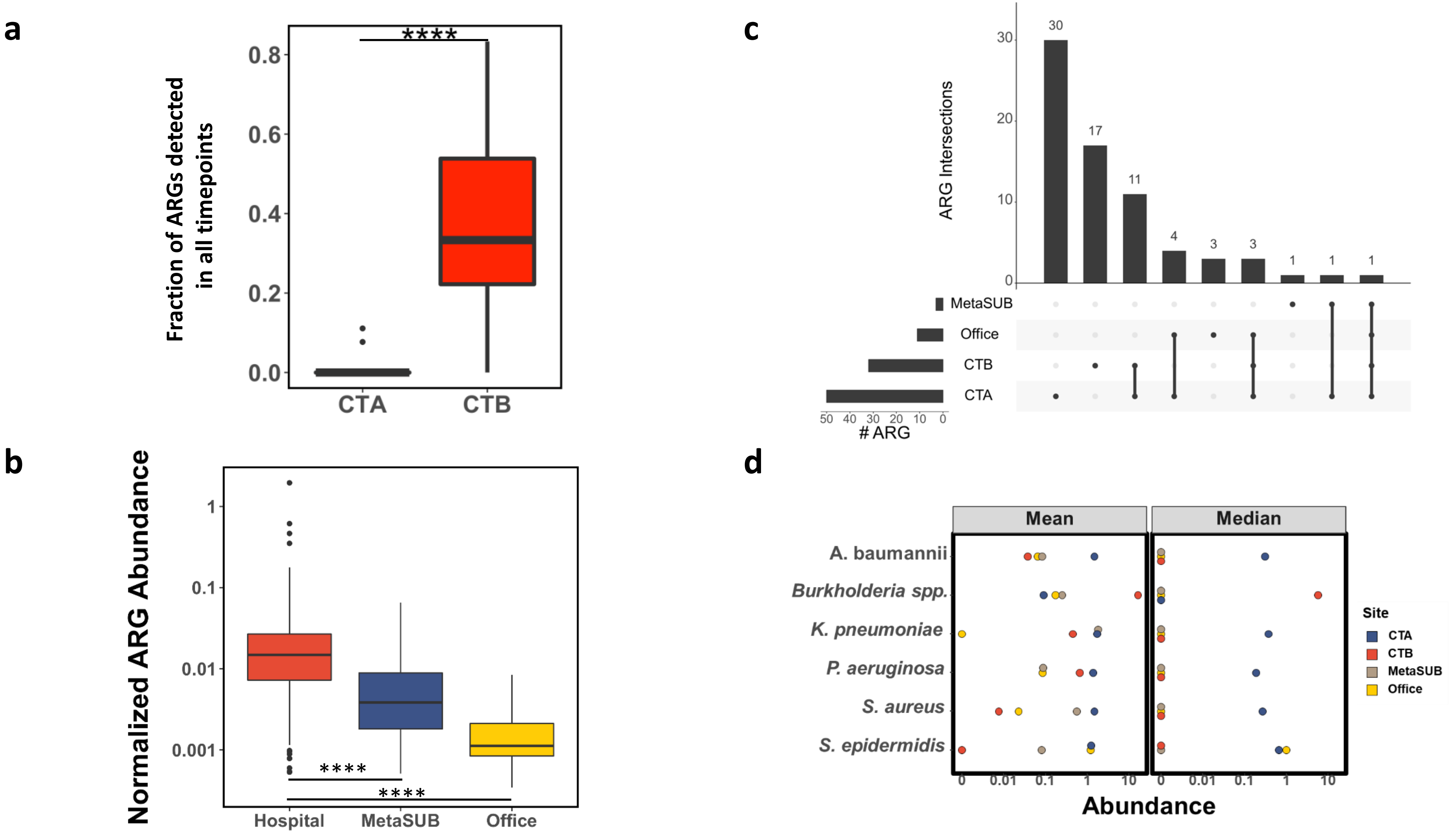

Supplementary Figure 7: a) Boxplots highlighting the stability of antibiotic resistance genes (ARGs) in CTB vs CTA sites (n=142 and 26 independent samples for CTA and CTB, respectively; two-sided Wilcoxon p-value<10^{-15}). b) Boxplots showing the dramatic enrichment of ARGs in hospital environments (y-axis on log-scale; n=427, 97 and 30 independent samples for hospital, MetaSUB and office sites, respectively; two-sided Wilcoxon p-value<10^{-15} for both tests). Boxplots in a) and b) are represented with center line: median; box limits: upper and lower quartiles; whiskers: 1.5× interquartile range; points: outliers 1.5× interquartile range away from the median. c) Upset plot showing overlaps in ARGs present in hospital (CTA or CTB sites), office and other environmental (MetaSUB; Singapore samples) microbiomes (normalized for sample size by subsampling; average of 100 replicates). d) Dotplots showing mean and median abundances of common nosocomial pathogens in the environment microbiomes of hospital (CTA or CTB), office and other community (MetaSUB) areas.

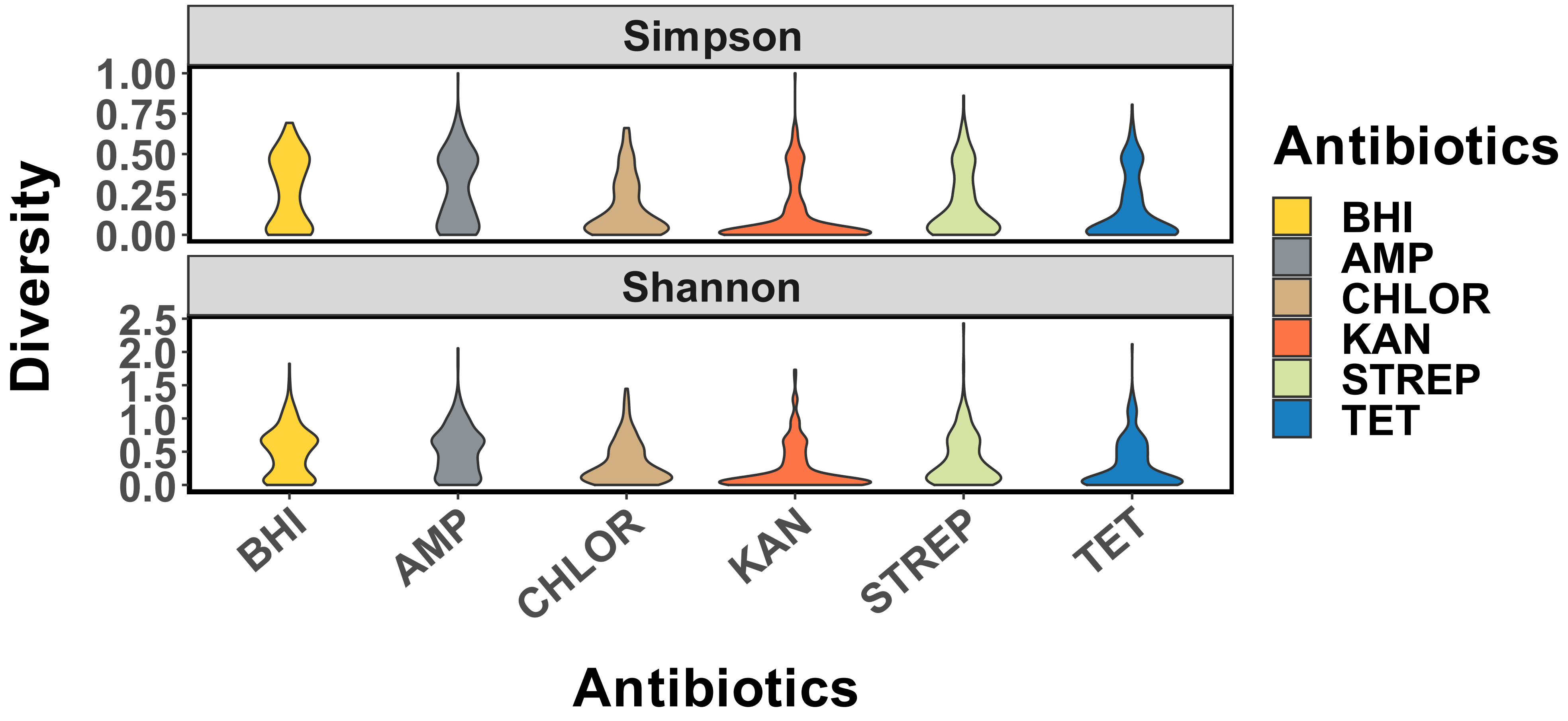

Supplementary Figure 8: Violin plots showing the distribution of genus-level diversity metrics for various culture-enriched communities (with BHI alone or BHI media supplemented with various antibiotics; n=292, 290, 199, 312, 220 and 288 independent samples for BHI, AMP, CHLOR, KAN, STREP and TET, respectively). The probability density of each violin plot was truncated at the minimum and maximum.

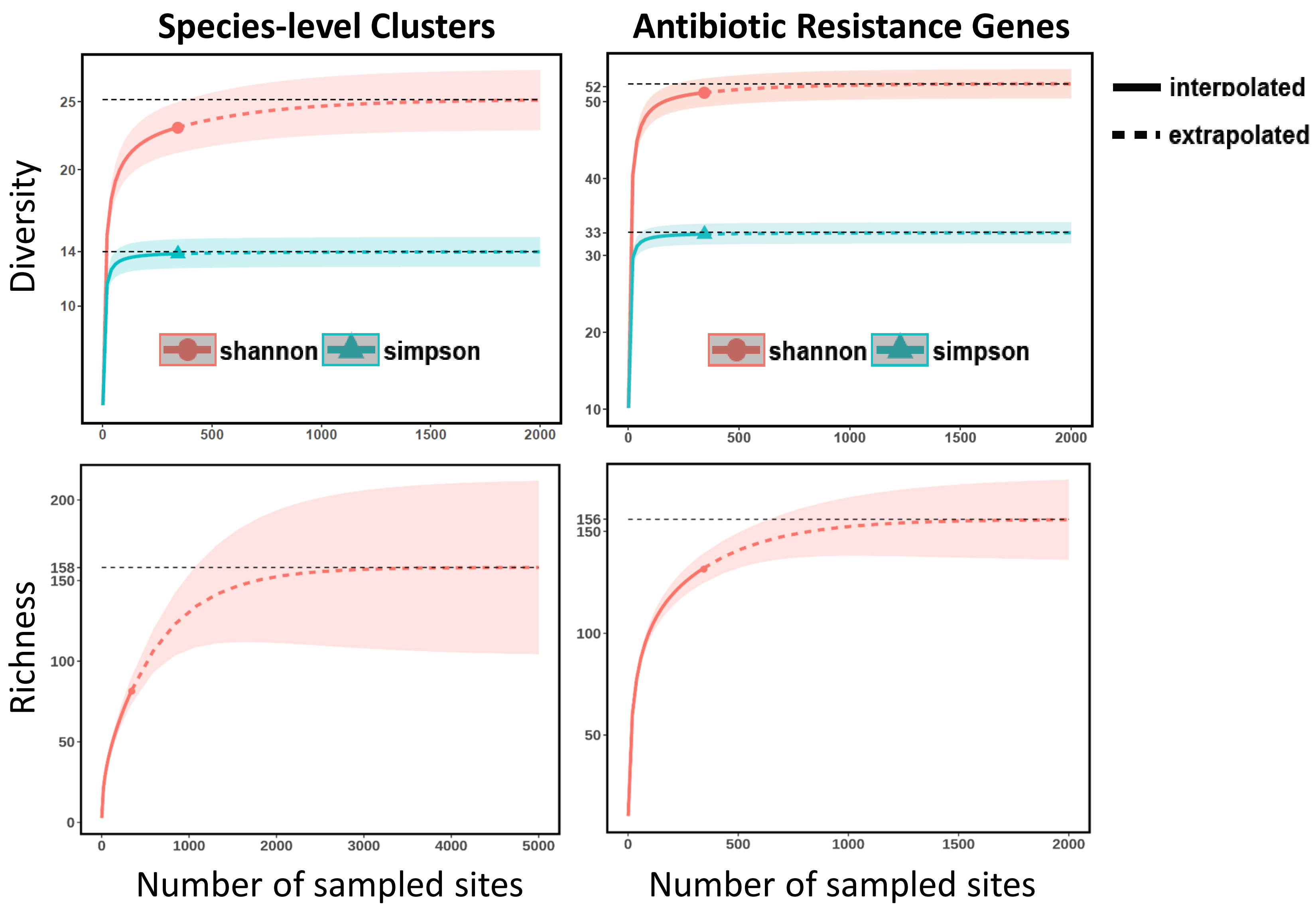

Supplementary Figure 9: Rarefaction analysis showing diversity (upper panel) and richness (lower panel) of species-level genomic clusters (ANI 95%) and antibiotic resistance genes observed in our genomic database as a function of the number of sites sampled. Current sampling efforts (triangle or circle) appear to capture >90% of the species and resistance gene diversity (>50% of richness) that can be sampled using this approach from the hospital environment microbiome. Shaded areas indicate 95% confidence intervals calculated based on n=356 samples.

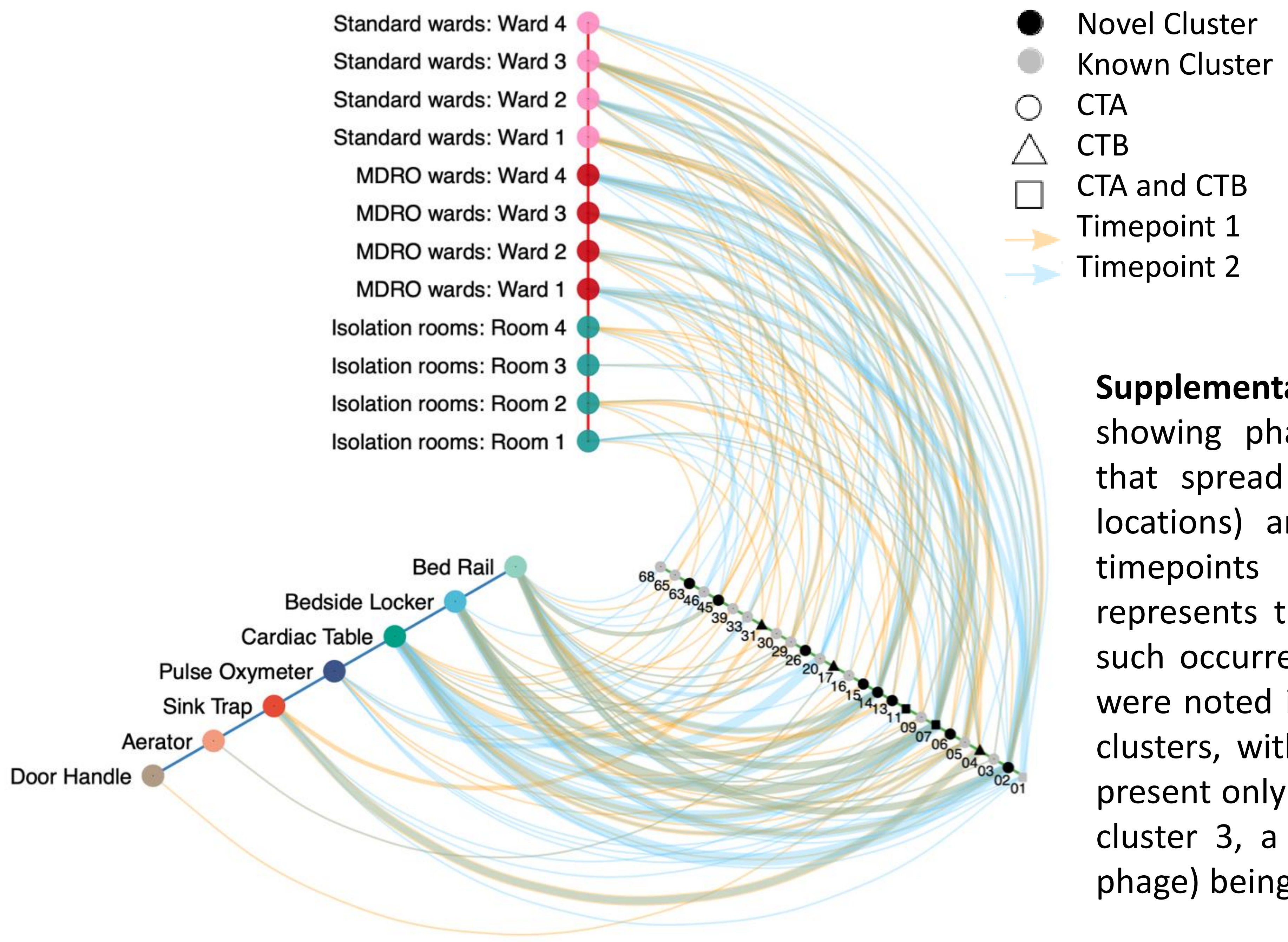

Supplementary Figure 10: Hive plot showing phage clusters (>99.9% ANI) that spread (observed at 2 or more locations) and/or persist (detected in timepoints 1 and 2). Line thickness represents the number of instances of such occurrences. Site-specific patterns were noted in the distribution of phage clusters, with most (77%, 20/26) being present only in CTA sites, and three (e.g. cluster 3, a novel telomere temperate phage) being present only in CTB sites.

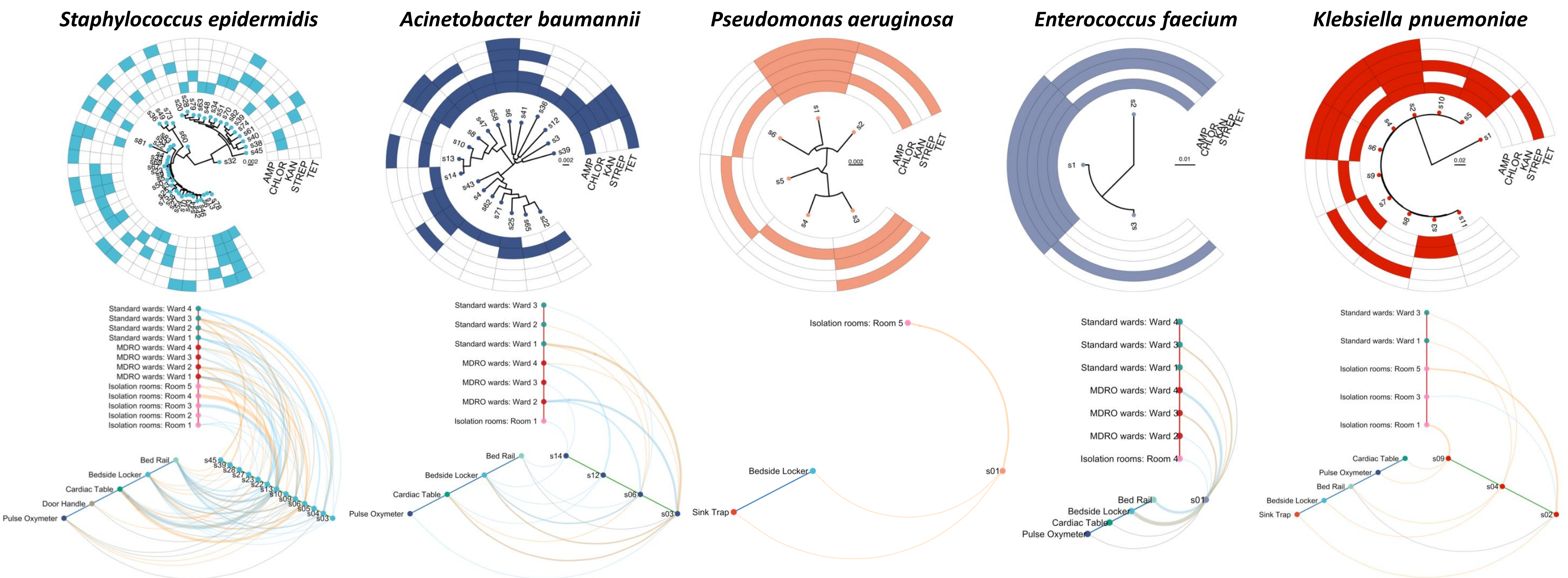

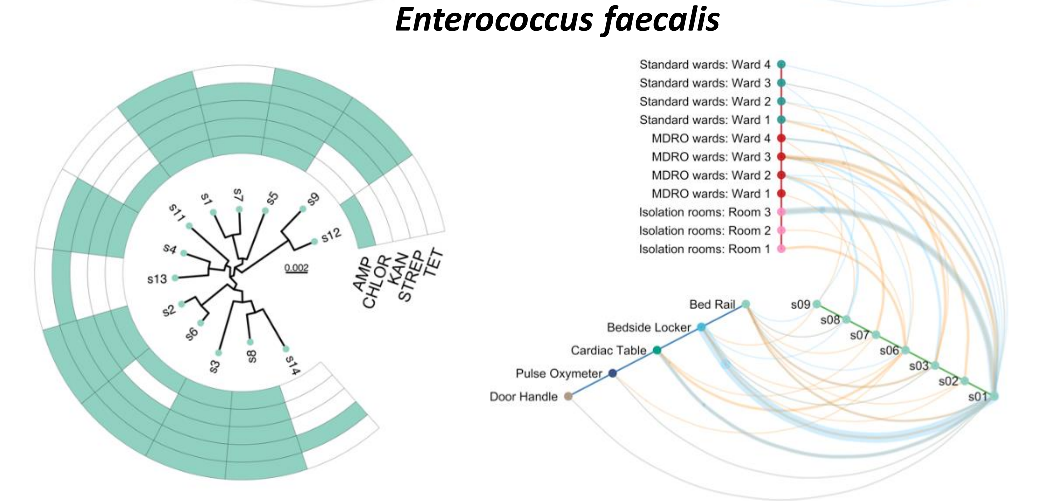

Supplementary Figure 11: Strain and derivative cluster phylogeny (>99.99% ANI for *S. epidermidis* and *A. baumannii* and >99.9% ANI otherwise; each leaf represents consensus genome of the cluster) of common nosocomial pathogens that were detected in the hospital environment with corresponding antibiotic resistance profiles, together with hive-map representation showing location of strains that spread (detected at 2 or more locations) and/or persist (detected at timepoints 1 and 2) in the hospital environment. The scale in each tree represents the number of substitutions per site, with respect to the core alignment. Orange lines represent occurrences at timepoint 1 while blue lines represent occurrences at timepoint 2. Line thickness represents the number of such observations that were made.

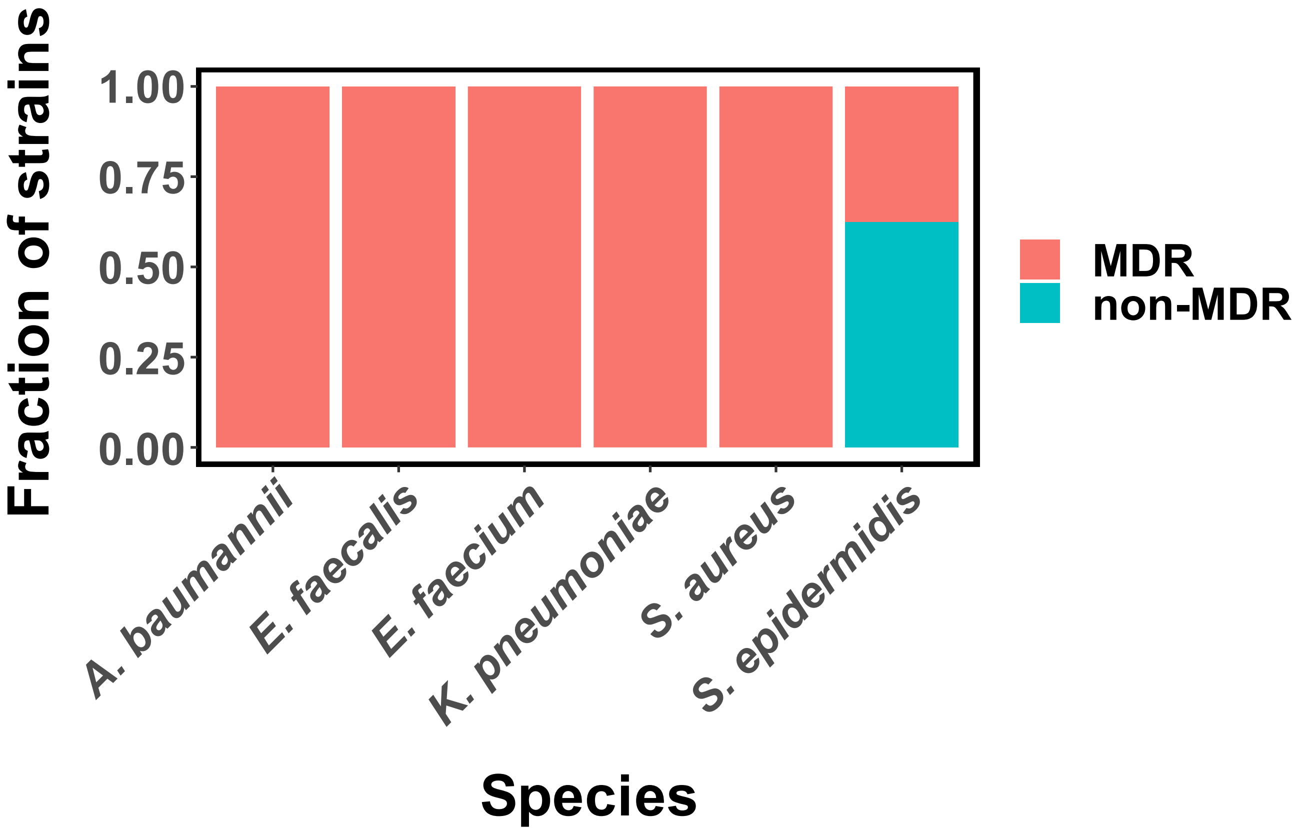

Supplementary Figure 12: Barplots showing the proportion of persistent (present in timepoints 1 and 2) strains (>99.99% ANI for *S. epidermidis, S. aureus* and *A. baumannii* and >99.9% ANI otherwise) that are multi-drug resistant (>2 antibiotics, MDR) for the different species.

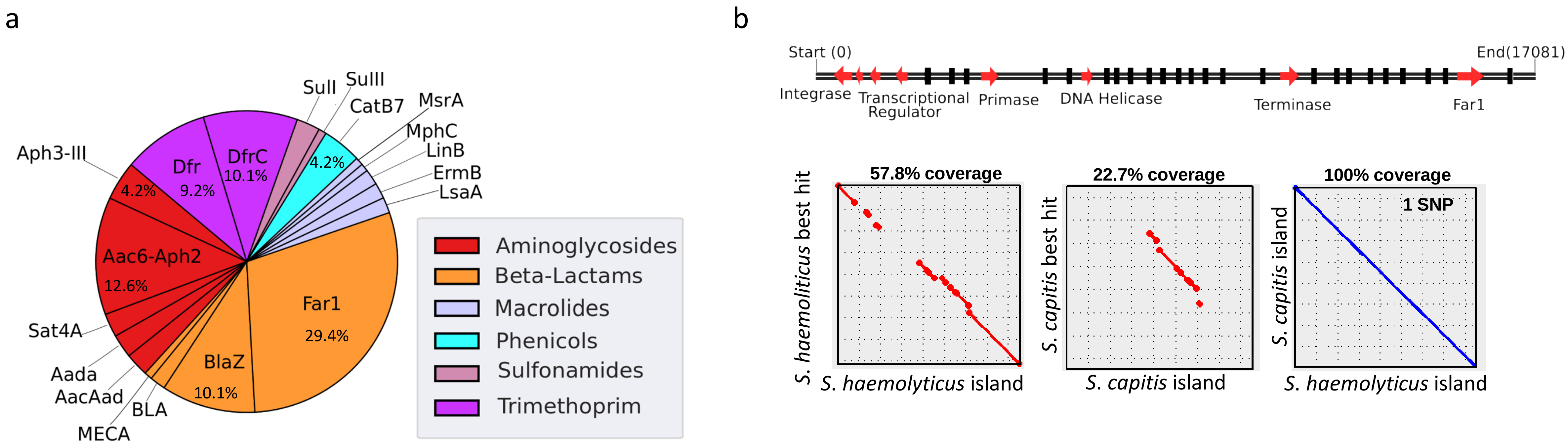

Supplementary 13: a) Pie chart showing the breakdown of antibiotic resistance genes in phages/prophages/phage-like-elements. Beta-lactam resistance genes were the dominant resistance class observed (42%) with Far1 being the most common resistance gene in this class (70%). b) (Top panel) Genome organization of a representative novel pathogenicity island from a phage-like element harboring the Far1 gene, observed in near identical copies in *S. haemolyticus* and *S. capitis* strains in the hospital environment (100% alignment, 99.994% ANI). Black lines represent hypothetical proteins and phage proteins. (Bottom Left and Center panel) Dotplots showing partial alignment between the novel pathogenicity island and its best blast hits (NCBI nt database) from *S. haemolyticus* and *S. capitis* respectively. (Bottom Right panel) Dotplot showing complete alignment between two representative pathogenicity islands found in *S. haemolyticus* and *S. capitis* cultured from the hospital environment, providing evidence for a transmission event mediated by a phage.

Supplementary Note 1: Assessing the impact of DNA contaminants on taxonomic profiles and identification of likely contaminant species

Following MetaSUB sample collection protocols[1], blank swabs exposed to air were collected in the hospital (handling controls) and in the laboratory environment where the samples were processed (laboratory controls). The amount of DNA extracted from handling controls was below detection limits for all swabs and hence DNA was pooled into 4 sets (from 4 samples each) for library preparation and sequencing. Comparison of hospital environment microbiomes with handling controls revealed that the taxonomic profiles observed in real samples were clearly distinct (across a range of biomass values), indicating that the impact of sampling and kitome contamination[2] on taxonomic profiles was limited (**Suppl. Note Fig. 1a**). This was further confirmed by sequencing of laboratory controls with spike-ins (*E. coli* cells and a Zymo mock community) at various concentrations (covering the range of samples that were processed in this study), where the spike-in samples exhibited very different profiles compared to blank laboratory controls (**Suppl. Note Fig. 1b**). Overall, DNA concentrations seen in libraries prepared from blank swabs were 250 to 25-fold lower than the amount seen with *E. coli* and Zymo spike-ins (3×10^5 cells), respectively.

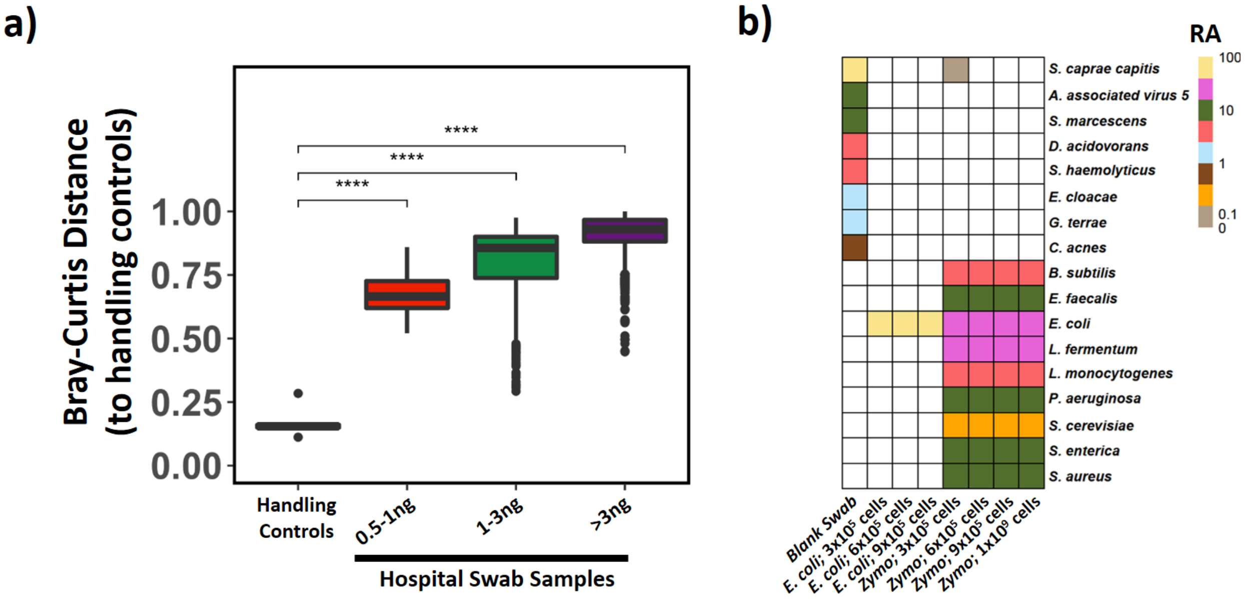

Supplementary Note Figure 1: a) Boxplots showing that handling controls have distinct taxonomic profiles from swabs collected in the hospital environment (genus-level Bray-Curtis dissimilarities) across a range of biomass values (n=6, 36, 469 and 888 combinations for handling controls, 0.5-1ng, 1-3ng and >3ng, respectively; two-sided Wilcoxon p-value=3.8×10^{-7}, 2.5×10^{-5} and 2.4×10^{-5} for handling control vs 0.5-1ng, 1-3ng and >3ng, respectively). More than 97% of hospital environment samples collected in this study have total DNA biomass >1ng (62% >3ng). Boxplots are represented with center line: median; box limits: upper and lower quartiles; whiskers: 1.5× interquartile range; points: outliers >1.5x interquartile range away from median. ****: p-value<0.0001. b) Heatmap showing species-level profiles of blank swab,

E. coli cells and a mock community (ZymoBIOMICS microbial community standard, Cat# D6300) controls for assessing the impact of the 'kitome' on taxonomic profiles of low biomass samples. Total extracted DNA biomass for the spike-ins are in parentheses: *E. coli* 3×10^5 cells (<0.1ng); 6×10^5 cells (1.2ng); 9×10^5 cells (1.8ng); mock community (Zymo) 3×10^5 cells (<0.1ng); 6×10^5 cells (<0.1ng); 9×10^5 cells (<0.1ng) and 1×10^9 cells (91ng DNA).

To additionally identify likely contaminant species we looked for discordance in prevalence across analysis batches[2] (timepoints 1 and 2 *versus* timepoint 3, which used different reagent kits and batches). Specifically, we identified species which were commonly present in one batch (>25% of samples with relative abundance >0.1%) but substantially less so in another (1/4th prevalence; red points in **Suppl. Note Fig. 2a**). The 7 species that were identified in this analysis also exhibited high correlation with each other (in 2 clusters of 5 and 2 taxa) as further evidence that they were likely contaminants[2] (**Suppl. Note Fig. 2b**). In addition, we confirmed that other potential contaminant species (close to thresholds used in **Suppl. Note Fig. 2a**) did not show high correlation with the 7 likely contaminant species (e.g. *Ruminococcus torques*, r<0.7) and/or were detected via culture based analysis (e.g. *Elizabethkingia anophelis*), and were therefore unlikely to be contaminants. A similar analysis was applied for ARGs and no genes were found to have a signature flagging them as being likely a function of laboratory contamination.

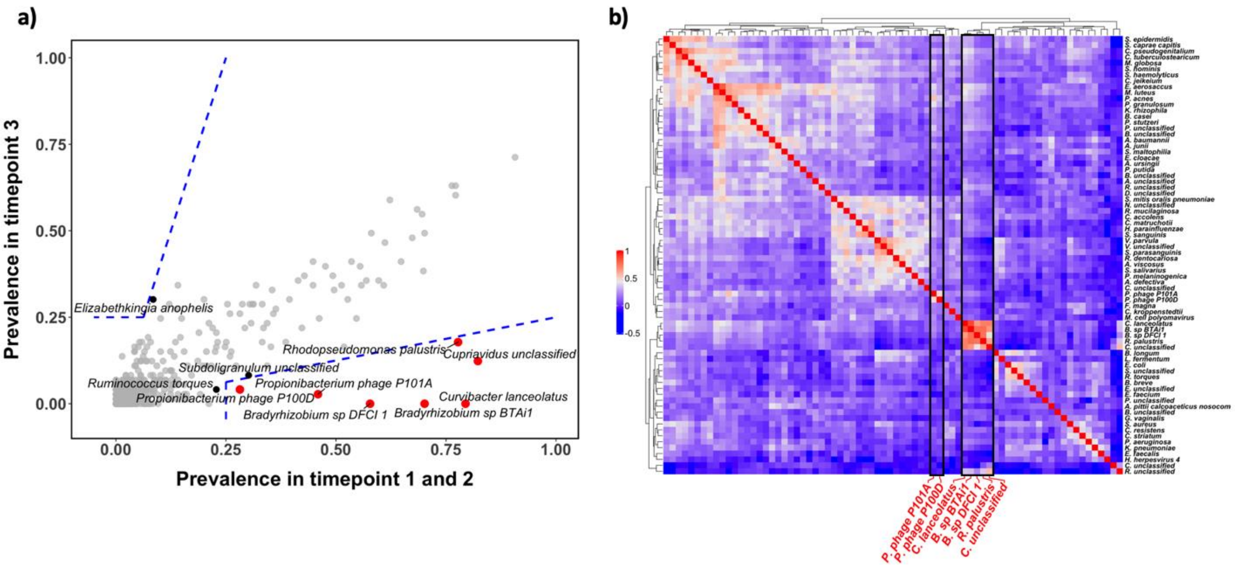

Supplementary Note Figure 2: a) Scatter plot showing the concordance of prevalence between batch 1 (timepoints 1 and 2) and batch 2 (timepoint 3) microbiomes (n=820 species). Thresholds used for identifying likely contaminant species are marked by blue lines and corresponding species are highlighted in red. Species that failed to meet the thresholds but were close (within 15%) are highlighted in black. b) Heatmap showing the correlation of abundances (Spearman) between species across samples in batch 1. Likely contaminant species are highlighted in red.

Supplementary Note 2: Validation of culturing and antibiotic based enrichment protocols

To test for the risk of contamination during culture-based enrichment, we tested 10 blank swabs as negative controls with the same culturing protocols as used for hospital environment swabs (**Online Methods**). All negative controls failed to exhibit growth, even after 48 hours of incubation, suggesting that the risk of contamination from the laboratory culturing process is low. We validated the effectiveness of the culture enrichment process for selecting antibiotic resistant microbes using 6 test swabs. For each sample after culture enrichment, microbes obtained from the 5 different types of antibiotic enrichment plates (Ampicillin, Chloramphenicol, Tetracycline, Kanamycin and Streptomycin sulfate) were streaked out onto 5 separate antibiotic-free BHI agar plates. After overnight incubation at 37˚C, we picked 10 colonies from each of the antibiotic-free BHI plates (5×10 colonies in total for 1 sample) and inoculated them separately into 100 µL of BHI broth supplemented with the antibiotic that was used originally for their enrichment (Ampicillin 100 µg/mL, Chloramphenicol 35 µg/mL, Kanamycin 50 µg/mL, Streptomycin sulfate 100 µg/mL, Tetracycline 10 µg/mL). Isolates that grew (high turbidity) after incubation at 37˚C overnight helped confirm antibiotic resistance. Only 3 out of 300 isolates (1%) did not exhibit the expected antibiotic resistance (**Supplementary Note Table 1**).

**Antibiotic Type**	**# of isolates exhibiting antibiotic resistance**					
	**Sample 1**	**Sample 2**	**Sample 3**	**Sample 4**	**Sample 5**	**Sample 6**
**Ampicillin**	10/10	10/10	10/10	10/10	10/10	10/10
**Chloramphenicol**	10/10	10/10	10/10	10/10	10/10	10/10
**Kanamycin**	9/10	10/10	10/10	10/10	10/10	10/10
**Streptomycin**	9/10	10/10	10/10	10/10	10/10	10/10
**Tetracycline**	10/10	10/10	10/10	10/10	10/10	9/10

Supplementary Note Table 1: Statistics for analysis confirming antibiotic resistance of isolates obtained from mixed cultures enriched with an antibiotic.

Supplementary Note 3: Rarefaction analysis for plasmids and strains

Rarefaction analysis for plasmids in our genomic database was used to estimate the overall diversity and richness that could have been captured. This analysis suggests that our current sampling captured >50% of the plasmid diversity (Shannon; clustered at 99% identity; 24% of richness) and a 10-fold increase in sampling (~4,000 samples) would be needed to capture the full diversity (**Supplementary Note Fig. 3a)**. Restricting the analysis to plasmids carrying antibiotic resistance genes improved sampling coverage only slightly (59% for diversity, **Supplementary Note Fig. 3b**), despite an almost complete sampling of resistance gene diversity (**Suppl. Fig. S9**). This is expected as plasmid genes can be highly mobile[3,4], consistent with the high diversity and plasticity of resistance gene combinations observed in our analysis (**Fig. 4**).

Rarefaction analysis of microbial strains also indicated that while our sampling was sufficient to reflect a majority of the strain diversity, an 8-fold increase in the size of the survey may be needed to get all strains of common nosocomial pathogens (**Supplementary Note Fig. 3c)**.

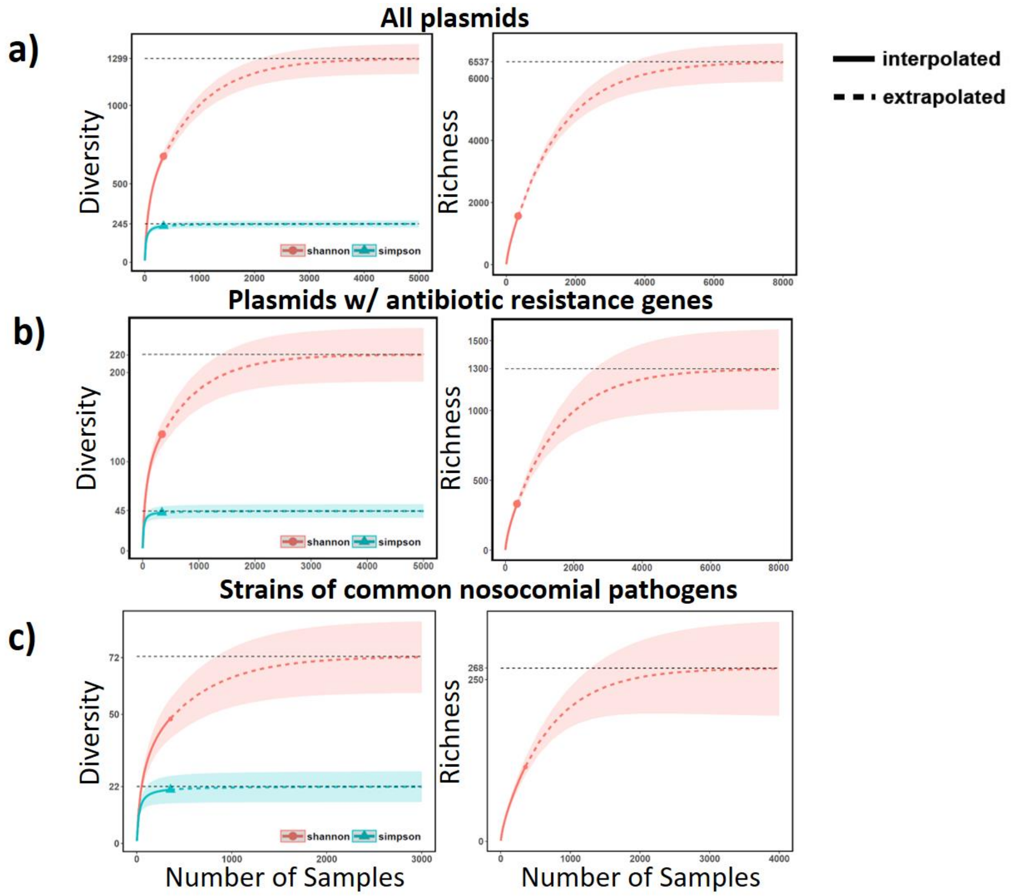

Supplementary Note Figure 3: Rarefaction analysis for plasmids and strains in the hospital environment. Shaded areas indicate 95% confidence intervals based on n=365 samples.

References

1 Danko, D. C. *et al.* Global Genetic Cartography of Urban Metagenomes and Anti-Microbial Resistance. *bioRxiv*, 724526, doi:10.1101/724526 (2019).

2 de Goffau, M. C. *et al.* Recognizing the reagent microbiome. *Nat Microbiol* **3**, 851-853, doi:10.1038/s41564-018-0202-y (2018).

3 Hall, J. P. J., Williams, D., Paterson, S., Harrison, E. & Brockhurst, M. A. Positive selection inhibits gene mobilisation and transfer in soil bacterial communities. *Nat Ecol Evol* **1**, 1348-1353, doi:10.1038/s41559-017-0250-3 (2017).

4 Fang, L. X. *et al.* High Genetic Plasticity in Multidrug-Resistant Sequence Type 3-IncHI2 Plasmids Revealed by Sequence Comparison and Phylogenetic Analysis. *Antimicrob Agents Chemother* **62**, doi:10.1128/AAC.02068-17 (2018).
